# Ethnopharmacological, Phytochemical, Pharmacological, and Toxicological Review on *Senna auriculata* (L.) Roxb.: A Special Insight to Antidiabetic Property

**DOI:** 10.3389/fphar.2021.647887

**Published:** 2021-08-24

**Authors:** Guruprasad C. Nille, Shardendu Kumar Mishra, Anand Kumar Chaudhary, K. R. C. Reddy

**Affiliations:** ^1^ Department of Rasa Shastra and Bhaishajya Kalpana, Faculty of Ayurveda, Institute of Medical Sciences, Varanasi, India; ^2^ Department of Pharmacology, Institute of Pharmacy, Ram-Eesh Institute of Vocational & Technical Education, Greater Noida, India

**Keywords:** *Cassia auriculata* L., ayurveda, antidiabetic, ethnomedicine, insulinogenic, Siddha, quercetin

## Abstract

Avartaki (*Senna auriculata* (L.) Roxb. syn. *Cassia auriculata* L.; Family- Fabaceae ) is a traditional medicinal plant, widely used for the treatment of various ailments in Ayurveda and Siddha system of medicine in India. Almost all the parts of the plant, such as flowers, leaves, seeds, barks, and roots have been reported for their medicinal uses. Traditionally, it has been used in the treatment of diabetes, asthma, rheumatism, dysentery, skin disease, and metabolic disorders. The principle phytochemicals in *Senna auriculata* (L.) Roxb. are alkaloids, anthraquinone, flavone glycosides, sugar, saponins, phenols, terpenoids, flavonoids, tannins, steroids, palmitic acid, linoleic acid, benzoic acid 2-hydroxyl methyl ester, 1-methyl butyl ester, resorcinol, α-tocopherol-β-D-mannosidase, epicatechin, ferulic acid, quercetin-3-O-rutinoside, quercetin, proanthocyanidin B1. The extracts from its different parts and their isolated compounds possess a wide range of pharmacological activities such as antidiabetic, antioxidant, anti-inflammatory, antihyperlipidemic, hepatoprotective, nephroprotective, cardioprotective, anti-atherosclerotic, anticancer, antimutagenic, antimicrobial, antiulcer, antipyretic, anthelmintic, immunomodulatory, antifertility, anti-venom, and anti-melanogenesis. The toxicological findings from preclinical studies ensured the safety of the plant, but comprehensive clinical studies are required for the safety and efficacy of the plant in humans. The current review article aimed to provide up-to-date information about *Senna auriculata* (L.) Roxb. covering its ethnomedicinal, phytochemical, pharmacological, and toxicological aspects with special emphasis on its clinical implications in diabetes.

## Introduction

The extensive use of medicinal plants to discover new therapeutics or active pharmacological compounds is the urgent need to tackle challenging non-communicable diseases (NCDs). Around 71% of the mortality worldwide has resulted due to NCDs, including the deaths of 40 million populations worldwide per year (Naghavi et al., 2017). In India alone, 60% of the deaths occur due to NCDs (Nethan et al., 2017). Diabetes and hypertension are the major contributory illnesses amidst the deaths caused by NCDs (Madavanakadu Devassy et al., 2020).

India has a great heritage of traditional system of medicines like Ayurveda, Siddha, and Unani, where hundreds of medicinal plants are being used to treat various diseases with their known ethnopharmacological evidence. *Senna auriculata* (L.) Roxb. syn. *Cassia auriculata* L., family Fabaceae (former Caesalpiniaceae) is one of the medicinal plants that has been used traditionally in Ayurveda, Siddha, and Unani since the 15th century. It is commonly known as Tanner’s Senna/Cassia and Mature Tea Tree in English; *Avartaki*, *Pitapuspa*, *Pitkalika*, *Manojyna*, *Pitkala*, *Charmaranga* in Sanskrit; *Tarwar*, *Awal*, *Tarval* in Hindi; *Tangedu*, *Merakatangeedu* in Telagu, and *Arsual*, *Taravada*, *Tarwad* in Marathi. This shrub is widely spread in India, covering its southern, westerns, and central dry zones and also in Sri Lanka (Gupta and Sharma, 2007; Nille and Reddy, 2017; Win and Min, 2018).

The current review aimed to investigate the ethnomedicinal uses, phytochemical, pharmacological, and toxicological studies of the *Senna auriculata* (L.) Roxb., and endeavored to validate the experimental studies in terms of scientific data concerning the therapeutic implications of active metabolites of the plant.

## Methodology

In the present review, the available literature has been explored from Ayurvedic classical texts, various published technical reports, online scientific data by accessing Scopus directory, Google Scholar, PubMed, Science Direct, EMBASE, SciFinder, Web of Science for its ethnomedicinal uses, phytochemistry, pharmacology, and biomedicinal applications. The following keywords were used: “*Senna auriculata* (L.) Roxb.,” “*Cassia auriculata* L.,” “Avartaki,” “Avarai,” “Tanner’s cassia,” “phytochemistry,” “antihyperglycemic,” “oxidative stress,” “antidiabetic,” “hepatoprotective,” “insulinogenic,” “anti-inflammatory,” “immunomodulator,” “antioxidant,” “antihyperlipidemic,” “mechanism of action” with their corresponding Medical Subject Headings (MeSH) terms using conjunctions OR/AND. The search was centered on identifying Ayurvedic claims in the available ethnomedicinal, phytochemical, pharmacological, clinical, and safety reports to understand the role of *Senna auriculata* (L.) Roxb. syn. *Cassia auriculata* L. in diabetes and other NCDs. The literature search was commenced before April 2021 and searches were limited to the English language.

## Botanical Aspects of *Senna auriculata* (L.) Roxb

*Senna auriculata* (L.) Roxb. is found in wooden grasslands up to a height of 500 m. It breeds wild in dry regions with annual precipitation of 300 mm. It grows well in areas with an annual temperature range of 15–28°C and needs full sun for its growth. It is a branched shrub with height of 1.5–5 m, trunk diameter of 20 cm and brown lenticellate bark (Kirtikar and Basu, 1918).

**FIGURE 1:**
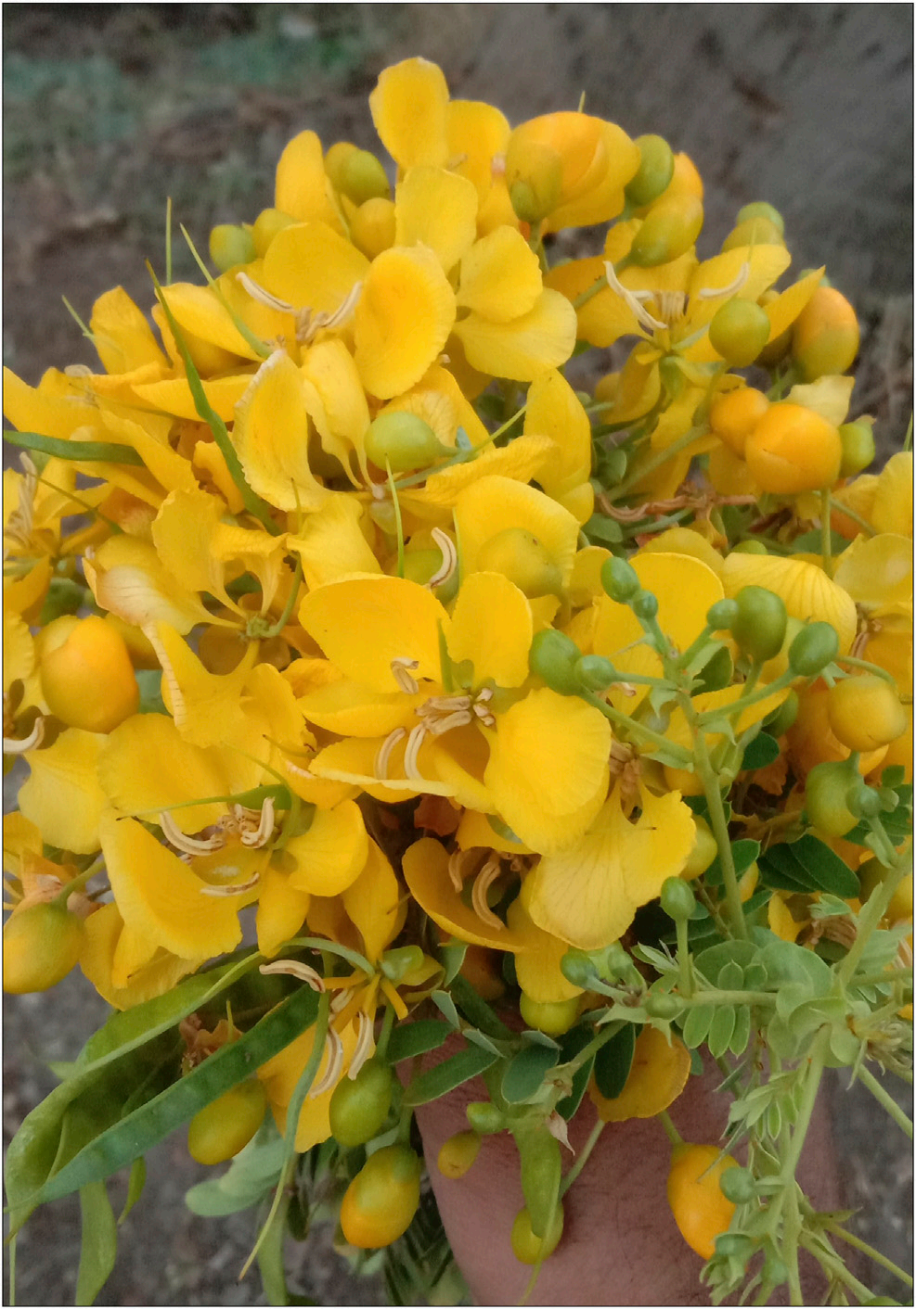
Flower & Leaves of *Senna auriculata* (L.) Roxb.

**Leaves:** Leaves are dull green in color, alternate, stipulate, paripinnate arrangement with 16–24 pairs of leaflets. Leaves are narrowly rough, pubescent and thin, with vertical and linear gland between the leaflets (Figure 1 **)**. It is short-stalked, 20–25 mm long, 10–13 mm wide; marginally overlapping, rectangular, dull-witted at both ends, and glabrous (Kirtikar and Basu, 1918; Evans, 1996).

**Flowers:** Flowers are bright yellow and irregular and large (50 mm) and have axillary raceme inflorescence, 2–8 flowered (Figure 1). Flowers are bisexual, zygomorphic, pentamerous, 4–5 cm; sepals are rounded at apex, imbricate; petals free, imbricate, unequal, 1.5–3 cm long; stamens 10, the 3 lower ones large stand fertile, others usually sterile; ovary superior, falcate, with 1.5 cm long, stalked, style (fruit a flattened) cylindrical pod 5–18 × 1–2 cm, transversely undulate between the 10–20 seeds, indehiscent, green turning to brown when mature (Kirtikar and Basu, 1918; Evans, 1996).

**FIGURE 2:**
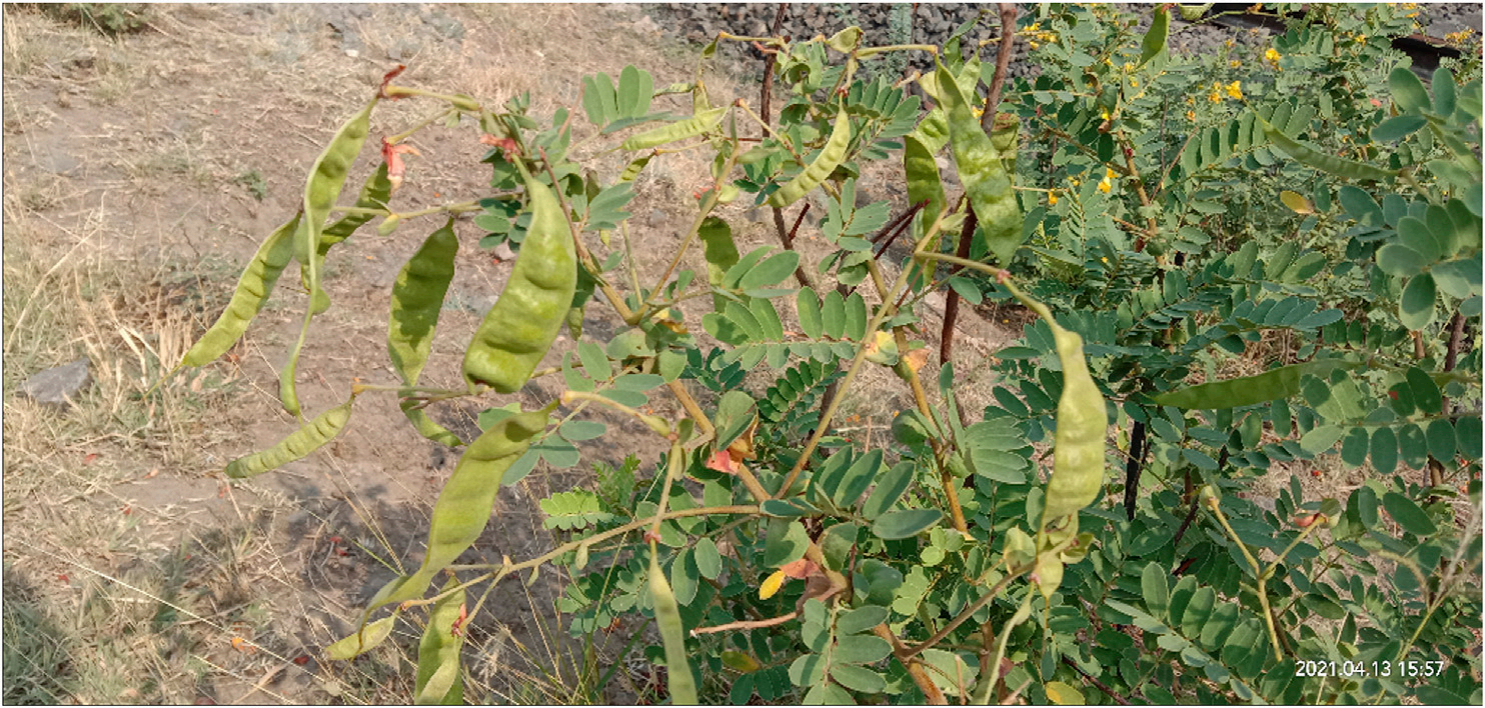
Fruits & Leaves of *Senna auriculata* (L.) Roxb.

**Fruits:** Fruits are green or light brown, and have legume which is 7–11 cm long, 1.5 cm wide, rectangular, long style base, flat, thin, undulate crimpled **(** Figure 2 **)**. It has about 12–20 seeds per fruit, each in its distinct cavity (Kirtikar and Basu, 1918; Evans, 1996).

## Traditional and Ethnopharmacological Uses of *Senna auriculata* (L.) Roxb

The foremost description of the *Senna auriculata* (L.) Roxb. is available in *Kaiyadeva Nighantu*, a classical Ayurveda text (15th century), where its *Pramehahara* property (antidiabetic action) through different botanical parts of the plant has been mentioned. Flowers have *Pramehashamana* property (antidiabetic action). Tender fruits have mentioned for their *Vamihara* (antiemetic), *Krimihara* (anthelmintic), *Sarvapramehahara* (antidiabetic), *Trishnaghna* (thirst alleviating), *Akshihita* (eye tonic), *Ruchya* (appetizing) properties. Seeds are useful as *Madhumehaghna* (antidiabetic), *Vishahara* (antidote), *Raktaatisaraghna* (anti-diarrheal). Roots are mentioned for *Trishnahara* (thirst alleviating), *Pramehaghna* (antidiabetic), *Shwasaghna* (antiasthamatic), *Raktapittashaman* (antihemorrhagic), *Shukrakshayahara* (sperm enhancing) properties (Sharma and Sharma, 1979). The leaf macerate of *Senna auriculata* (L.) Roxb. was used traditionally to treat inflammation specifically in Maharashtra, Andhra Pradesh, and Gujarat. It also effectively reduces pain and inflammation in joint disorders. The leaves of *Senna auriculata* (L.) Roxb. are also effective in muscle pain, irregular muscle contraction, body pain, gastritis, skin sores and ulcers. The flowers are effectively used as health beneficial agents. The crushed flowers are mixed with goat milk to cure sexually transmitted diseases. The dried powder of flowers of *Senna auriculata* (L.) Roxb. is used to clean the hair, and taken by diabetic patients and in fever. The root is used by chewing, and the juice is swallowed to cure abdominal complaints, vomiting, urinary discharges, tumors and diarrhea. Powder of bark is used to treat toothache by applyingit to the gums. The fruits are used in helminthic infections (Punjani, 2002; Punjani and Kumar, 2002; Duraipandiyan et al., 2006; Pawar and Patil, 2007; Ignacimuthu et al., 2008; Kosalge and Fursule, 2009; Thirumalai et al., 2009; Patel et al., 2010; Jagatheeswari, 2012).

## Metabolites of *Senna auriculata* (L.) Roxb

The flower, leaves, roots and seed were investigated with advanced spectroscopic and chromatographic techniques and found various active metabolites with therapeutic implications against different diseases. The flowers of *Senna auriculata* (L.) Roxb. reported a significant amount of alkaloids, glycosides, saponins, polyphenols, tannins, phloro-tannins, terpenoids, triterpenes, carbohydrates, proteins, amino acids, anthraquinone, aloe-emodin, and sitosterols. These metabolites attributed towards the pharmacological action in diabetes mellitus and other ailments.

Leaves of *Senna auriculata* (L.) Roxb. shows the presence of 3-o-methy-d glucose, alpha-tocopherol–beta–D –mannosidase, n-hexadecanoic acid, resorcinol, octadecenal and carboxylic acid. The seeds of *Senna auriculata* (L.) Roxb. reported with presence of fatty acids content are palmitic, oleic, and linoleic acids. The ethanolic seed extract showed the presence of benzoic acid, 2-hydroxyl methyl ester, glycine, n-(trifluroacetyl), 1-methybutul ester, cupric acid ethyl ester, resorcinol, water-soluble galactomannan like beta-D-manopyranosyl-1(1–4)-o-beta-D-manopyronosyl (1–4)-o-beta–D-monopyranose. The reported metabolites have potential role in oxidative stress, microbial infections and inflammation related diseases.

**FIGURE 3:**
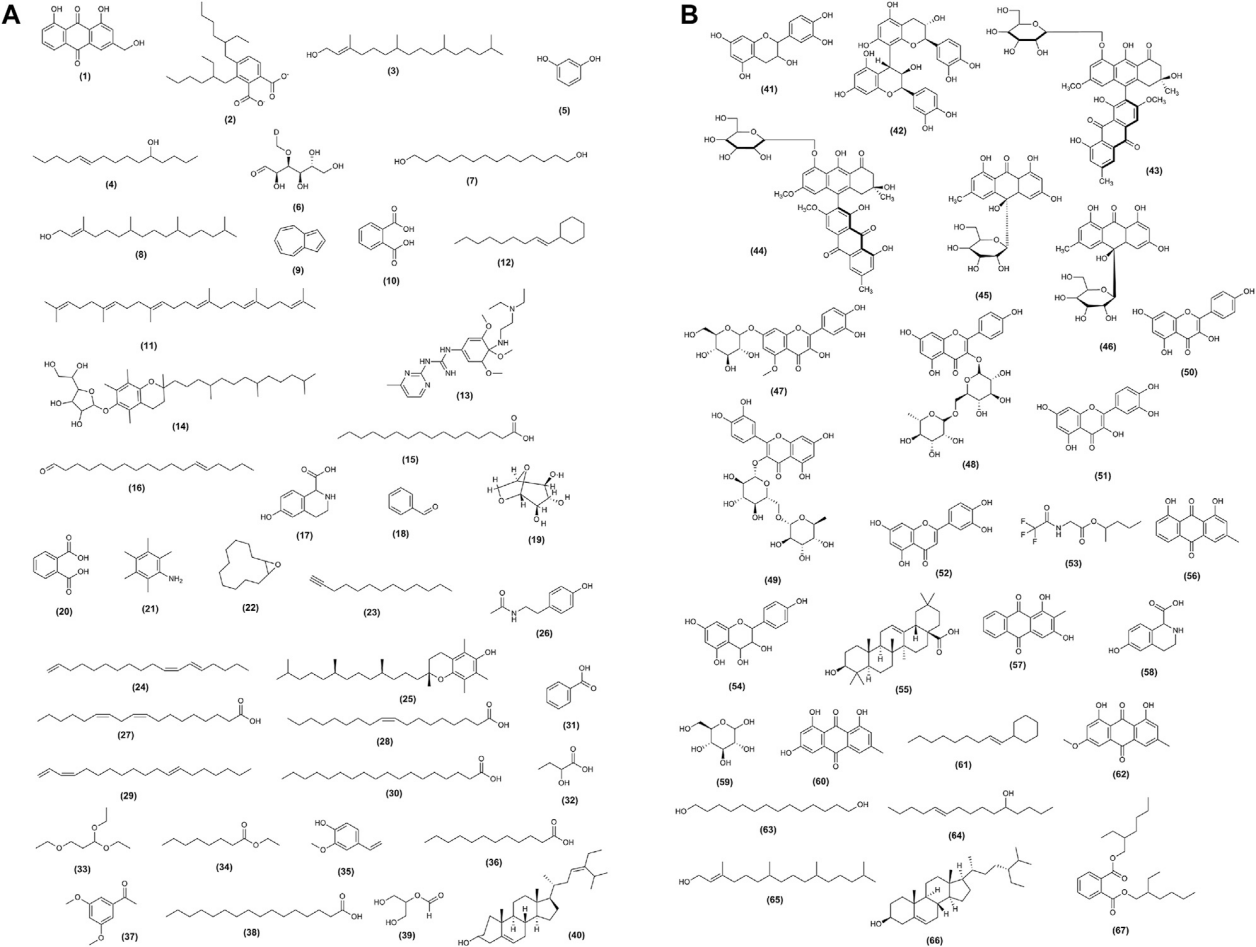
**(A)** Structure of metabolites of *Senna auriculata* (L.) Roxb. **(B)** Structure of metabolites of *Senna auriculata* (L.) Roxb.

Roots of *Senna auriculata* (L.) Roxb. shows the presence of anthraquinone glycosides such as 1,3-dihydroxy-2-methylanthraquinone, 1,8-dihydroxy-6-methoxy-2-methylanthraquinone-3-o-rutinoside, 1,8-dihydroxy-2-methylanthraquinone-3-o-rutinoside, flavone glycoside, and two leucoanthocyanins like leucocyanidin-3-o-rhamnopyroside and leucopeonidin-3-o-1-rhamanopyroside. The list of reported metabolites is enlisted in Table 1 and Figures 3A,B.

**TABLE 1:** Metabolites of *Senna auriculata* (L.) Roxb.

S No.	Name of metabolites	Isolated from a plant part	References
1.	Emodin**(1)**	Leaves	Nageswara Rao et al. (2000)
2.	Di-(2-ethyl)-hexylphthalate**(2)**	Leaves	Nageswara Rao et al. (2000)
3.	Phytol**(3)**	Leaves	Senthilkumar and Vijayakumari (2012)
4.	*E -10-* pentadecenol**(4)**	Leaves	Senthilkumar and Vijayakumari (2012)
5.	Resorcinol **(5)**	Leaves	Senthilkumar and Vijayakumari (2012)
6.	3-*O*-methyl-d-glucose **(6)**	Leaves	Senthilkumar and Vijayakumari (2012), Anandan et al. (2011)
7.	1,14-tetradecanediol **(7)**	Leaves	Senthilkumar and Vijayakumari (2012)
8.	3,7,11,15- tetramethyl-2-hexadecen-1-ol **(8)**	Leaves	Senthilkumar and Vijayakumari (2012)
9.	Azulene**(9)**	Leaves	Senthilkumar and Vijayakumari (2012)
10.	1,2,3,5,6,7,8,8a-octahydro-1,4-dimethyl-7-(1-methylethenyl)	Leaves	Senthilkumar and Vijayakumari (2012)
11.	1,2- benzene dicarboxylic acid **(10)**	Leaves	Senthilkumar and Vijayakumari (2012)
12.	Diisooctyl ester	Leaves	Senthilkumar and Vijayakumari (2012)
13.	Squalene**(11)**	Leaves	Senthilkumar and Vijayakumari (2012)
14.	1-cyclohexylnonene **(12)**	Leaves	Senthilkumar and Vijayakumari (2012)
15.	1–4 - [(2- diethylamino] ethylamino[-6-methyl-2-pyrimidinyl]-3- [3,4,5- trimethoxyphenyl] guanidine **(13)**	Leaves	Senthilkumar and Vijayakumari (2012)
16.	α-tocopherol-β- d -mannoside**(14)**	Leaves	Anandan et al. (2011)
17.	*n*-hexadecanoic acid **(15)**	Leaves	Anandan et al. (2011)
18.	13-octadecenal **(16)**	Leaves	Anandan et al. (2011)
19.	1,2,3,4-tetrahydroisoquinolin-6-ol-1-carboxylic acid **(17)**	Leaves	Anandan et al. (2011)
20.	Benzaldehyde**(18)**	Leaves	Anandan et al. (2011)
21.	1,6-anhydro-β-d-glucopyranose **(19)**	Leaves	Anandan et al. (2011)
22.	1,2-benzenedicarboxylic acid **(20)**	Leaves	Anandan et al. (2011)
23.	Bis (2-methylpropyl) ester	Leaves	Anandan et al. (2011)
24.	Benzenamine,2,3,4,5,6- pentamethyl**(21)**	Leaves	Anandan et al. (2011)
25.	13-oxabicyclo [10.1.0] tridecane**(22)**	Leaves	Anandan et al. (2011)
26.	1-tridecyne **(23)**	Leaves	Anandan et al. (2011)
27.	1-*E*,11,*Z-*13-octadecatriene **(24)**	Leaves	Anandan et al. (2011)
28.	*a* -tocopherol**(25)**	Leaves	Anandan et al. (2011)
29.	*N* -acetyl tyramine**(26)**	Leaves	Anandan et al. (2011)
30.	*n* -hexadecanoic acid **(15)**	Seed/Pod	Suresh et al. (2007), Raj et al. (2012), Puranik et al. (2011)
31.	Linoleic acid **(27)**	Seed/Pod	Suresh et al. (2007), Raj et al. (2012), Puranik et al. (2011)
32.	Oleic acid **(28)**	Seed/Pod	Suresh et al. (2007), Raj et al. (2012), Puranik et al. (2011)
32.	*E*, *Z*-1,3,12-nonadecatriene **(29)**	Seed/Pod	Suresh et al. (2007), Raj et al. (2012), Puranik et al. (2011)
34.	Stearic acid **(30)**	Seed/Pod	Suresh et al. (2007), Raj et al. (2012), Puranik et al. (2011)
35.	Benzoic acid **(31)**	Seed/Pod	Suresh et al. (2007), Raj et al. (2012), Puranik et al. (2011)
36.	2-hydroxyl-methyl ester **(32)**	Seed/Pod	Suresh et al. (2007), Raj et al. (2012), Puranik et al. (2011)
37.	β-ethoxypropionaldehyde diethyl acetal**(33)**	Seed/Pod	Suresh et al. (2007), Raj et al. (2012), Puranik et al. (2011)
38.	Ethyl caprylate**(34)**	Seed/Pod	Suresh et al. (2007), Raj et al. (2012), Puranik et al. (2011)
39.	2-methoxy-4-vinylphenol **(35)**	Seed/Pod	Suresh et al. (2007), Raj et al. (2012), Puranik et al. (2011)
40.	*N* -(trifluoroacetyl)-,1-methylbutyl ester	Seed/Pod	Suresh et al. (2007), Raj et al. (2012), Puranik et al. (2011)
41.	2,3-dihydro-3,5-dihydroxy-6-methyl- 4 *H* - pyran-4-one	Seed/Pod	Suresh et al. (2007), Raj et al. (2012), Puranik et al. (2011)
42.	Dodecanoic acid **(36)**	Seed/Pod	Suresh et al. (2007), Raj et al. (2012), Puranik et al. (2011)
43.	3′5′-dimethoxyacetophenone **(37)**	Seed/Pod	Suresh et al. (2007), Raj et al. (2012), Puranik et al. (2011)
44.	Palmitic acid **(38)**	Seed/Pod	Suresh et al. (2007), Raj et al. (2012), Puranik et al. (2011)
45.	β-monoglyceride**(39)**	Seed/Pod	Suresh et al. (2007), Raj et al. (2012), Puranik et al. (2011)
46.	α-tocopherol**(25)**	Seed/Pod	Suresh et al. (2007), Raj et al. (2012), Puranik et al. (2011)
47.	Stigmasta-5,23-dien-3-ol **(40)**	Seed/Pod	Suresh et al. (2007), Raj et al. (2012), Puranik et al. (2011)
48.	Epicatechin**(41)**	Seed/Pod	Suresh et al. (2007), Raj et al. (2012), Puranik et al. (2011)
49.	Procyanidin B1 **(42)**	Seed/Pod	Suresh et al. (2007), Raj et al. (2012), Puranik et al. (2011)
50.	Auriculataosides-A **(43)**	Seed/Pod	Wang et al. (2019)
51.	Auriculataosides- B **(44)**	Seed/Pod	Wang et al. (2019)
52.	Rumejaposides-E **(45)**	Seed/Pod	Wang et al. (2019)
53.	Rumejaposides-F **(46)**	Seed/Pod	Wang et al. (2019)
54.	5-*O*-methylquercetin-7-*O*-glucoside **(47)**	Flower	Manogaran and Sulochana (2004)
55.	Propanoic acid	Flower	Venkatachalam et al. (2013)
56.	Kaempferol-3-*O*-rutinoside **(48)**	Stem	Juan-Badaturuge et al. (2011), Habtemariam (2013)
57.	Rutin**(49)**	Stem	Juan-Badaturuge et al. (2011), Habtemariam, (2013)
58.	Kaempferol**(50)**	Stem	Juan-Badaturuge et al. (2011), Habtemariam (2013)
59.	Quercetin **(51)**	Stem	Juan-Badaturuge et al. (2011), Habtemariam (2013)
60.	Luteolin**(52)**	Stem	Juan-Badaturuge et al. (2011), Habtemariam (2013)
61.	Glycine,*N*-(trifluoroacetyl)-, 1-methylbutyl ester **(53)**	Stem	Juan-Badaturuge et al. (2011), Habtemariam (2013), Suresh et al. (2007), Raj et al. (2012), Puranik et al. (2011)
62.	Leucopelargonidins**(54)**	Aerial Part	Juan-Badaturuge et al. (2011), Habtemariam (2013), Suresh et al. (2007), Raj et al. (2012), Puranik et al. (2011), Manogaran and Sulochana, (2004)
63.	Oleanolic acid **(55)**	Aerial Part	Juan-Badaturuge et al. (2011), Habtemariam (2013), Suresh et al. (2007), Raj et al. (2012), Puranik et al. (2011), Manogaran and Sulochana, (2004)
64.	Chrysophanol**(56)**	Aerial Part	Juan-Badaturuge et al. (2011), Habtemariam (2013), Suresh et al. (2007), Raj et al. (2012), Puranik et al. (2011), Manogaran and Sulochana, (2004)
65.	1,3-dihydroxy-2-methylanthraquinone **(57)**	Aerial Part	Juan-Badaturuge et al. (2011), Habtemariam (2013), Suresh et al. (2007), Raj et al. (2012), Puranik et al. (2011), Manogaran and Sulochana, (2004)
66.	1,2,3,4-Tetrahydroisoquinolin-6-ol-1-carboxylic acid **(58)**	Aerial Part	Juan-Badaturuge et al. (2011), Habtemariam (2013), Suresh et al. (2007), Raj et al. (2012), Puranik et al. (2011), Manogaran and Sulochana, (2004)
67.	d – glucopyranoside**(59)**	Aerial Part	Juan-Badaturuge et al. (2011), Habtemariam, (2013), Suresh et al. (2007), Raj et al. (2012), Puranik et al. (2011), Manogaran and Sulochana (2004)
68.	1,6,8-trihydroxy-3-methylanthraquinone **(60)**	Aerial Part	Juan-Badaturuge et al. (2011), Habtemariam (2013), Suresh et al. (2007), Raj et al. (2012), Puranik et al. (2011), Manogaran and Sulochana, (2004)
69.	1-cyclohexylnonene **(61)**	Aerial Part	Juan-Badaturuge et al. (2011), Habtemariam, (2013), Suresh et al. (2007), Raj et al. (2012), Puranik et al. (2011), Manogaran and Sulochana (2004)
70.	1,8-dihydroxy-3-methoxy-6-methylanthracene-9,10-dione **(62)**	Aerial Part	Juan-Badaturuge et al. (2011), Habtemariam, (2013), Suresh et al. (2007), Raj et al. (2012), Puranik et al. (2011), Manogaran and Sulochana (2004)
71.	1,14-tetradecanediol **(63)**	Aerial Part	Juan-Badaturuge et al. (2011), Habtemariam, (2013), Suresh et al. (2007), Raj et al. (2012), Puranik et al. (2011), Manogaran and Sulochana (2004)
72.	E -10-pentadecenol **(64)**	Aerial Part	Juan-Badaturuge et al. (2011), Habtemariam (2013), Suresh et al. (2007), Raj et al. (2012), Puranik et al. (2011), Manogaran and Sulochana (2004)
73.	3,7,11,15-tetramethyl-2-hexadecen-1-ol **(65)**	Aerial Part	Juan-Badaturuge et al. (2011), Habtemariam (2013), Suresh et al. (2007), Raj et al. (2012), Puranik et al. (2011), Manogaran and Sulochana (2004)
74.	Sitosterol**(66)**	Aerial Part	Juan-Badaturuge et al. (2011), Habtemariam (2013), Suresh et al. (2007), Raj et al. (2012), Puranik et al. (2011), Manogaran and Sulochana (2004)
75.	Di-(2-ethyl) hexylphthalate**(67)**	Aerial Part	Juan-Badaturuge et al. (2011), Habtemariam (2013), Suresh et al. (2007), Raj et al. (2012), Puranik et al. (2011), Manogaran and Sulochana (2004)

## Pharmacological Actions of *Senna auriculata* (L.) Roxb

### Antidiabetic Activity

#### Antihyperglycemic Activity of Plant Parts of *Senna auriculata* (L.) Roxb

##### Leaves

Antihyperglycemic activity of *Senna auriculata* (L.) Roxb. leaves extract has experimentally been proven in different diabetic animal models (Rajagopal et al., 2003; Gupta et al., 2009a; Gupta et al., 2009b; Gupta et al., 2010). The aqueous extract of leaves of *Senna auriculata* (L.) Roxb. is reported to reduce the blood glucose levels in STZ induced diabetic rats (Gupta et al., 2011). Further, Gupta et al. (2009c) reported the potential antihyperglycemic effects of STZ induced mild and severely diabetic rats on the administration of the aqueous extract of leaves in 400 mg/kg body weight dose for 3 weeks. The 13.9% and 17.4% reduction in fasting blood glucose was observed after 5 h of dose administration in mild and severely diabetic rats respectively. The ethanolic extract of *Senna auriculata* (L.) Roxb. leaf showed significant reduction of blood glucose (*p* < 0.05) at a dose of 150 mg/kg of body weight for 2 weeks in alloxan induced diabetic rats. The results were compared with a standard drug, glibenclamide (Mohan et al., 2011).

The leaves of *Senna. auriculata* (L.) Roxb has various antidiabetic metabolites which are enlisted in Table 1. n-hexadecanoic acid, emodin (1,3,8-trihydroxy-6-methylanthracene-9,10-dione), and squalene are evaluated for their antidiabetic potential and reported with their mechanistic pathways involved in the amelioration of diabetes (Wang et al., 2006; Wang et al., 2012; Liu et al., 2018; Tan et al., 2019). These metabolites might be responsible for the antihyperglycemic effects of leaves of *Senna auriculata* (L.) Roxb.

##### Flower

Aqueous extract of *Senna auriculata* (L.) Roxb. flower has shown promising antihyperglycemic activity in a dose 450 mg/kg body weight which found more significant than the doses of 150 and 300 mg/kg body weight. Similarly, the significant reduction in the urine sugar was found with aqueous extract of *Senna auriculata* (L.) Roxb. at doses of 150 and 300 mg/kg body weight whereas, at 450 mg/kg dose, sugar was absent in the urine of experimental diabetic rats. The result was more promising than glibenclamide-treated rats (Latha and Pari, 2003a; Latha and Pari, 2003b). Also, Jain and Sharma, (1997) reported the anti-diabetic potential of the aqueous flower extract of *Senna auriculata* (L.) Roxb.

The ethanol extract of *Senna auriculata* (L.) Roxb. flower possesses antidiabetic agents such as flavonoids and phenolic acids. The anti-diabetic activity of water-soluble fraction of the ethanol extract was compared with aqueous extract of the flower where it was found that ethanol extract exhibited a more significant reduction in blood glucose level at a dose of 250 and 500 mg/kg of the body weight in alloxan-induced diabetic rats. The highly significant (*p* < 0.001) results of ethanol extract of the flower might be due to the metabolites present in the water-soluble fraction of the ethanolic extract (Hakkim et al., 2007). Similarly, it is observed that the ethanolic extract exhibits anti-diabetic activity and provides significant protection in streptozotocin-nicotinamide induced diabetic rats (*p* < 0.05) and the results were statistically significant in comparison to the standard glibenclamide (Nalla et al., 2012).

Jarald et al. (2010) performed an experimental study with various extracts of flowers of *Senna auriculata* (L.) Roxb. in alloxan-induced diabetic rats to check their anti-hyperglycemic activities. Amongst different extracts, the chloroform extract was reported to have more hypoglycemic effects followed by the water-soluble fraction of ethanolic extract and ethanolic extract. The results found comparable with the standard drug, glibenclamide.

Various active compounds from the flowers of *Senna auriculata* (L.) Roxb. have been isolated and their potential antidiabetic activity has been reported with experimental evidence. Venkatachalam et al. (2013) reported the presence of propanoic acid, 2-(3-acetoxy-4,4,14-trimethylandrost-8-en-17-yl) as an active principle of the n-butanol fraction of hydromethonolic flower extract of *Senna auriculata* (L.) Roxb. Propanoic acid showed potential hypoglycemic effects both in the bioassay-guided study as well as *in vivo* study, due to its protein tyrosine phosphatase 1B (PTP 1B) inhibitory action. The isolated phytochemical compound exerted comparable result as that of standard drug, glibenclamide. Mishra et al. (2010) and Jyothi et al. (2012) have also demonstrated the anti-diabetic potential of flowers of *Senna auriculata* (L.) Roxb. Oleanolic acid, a natural triterpenoid present in the aerial part of Senna auriculata (L.) Roxb. (Manogaran and Sulochana, 2004; Suresh et al., 2007; Juan-Badaturuge et al., 2011; Puranik et al., 2011; Raj et al., 2012; Habtemariam, 2013) exhibited a significant blood-glucose-lowering and weight-losing activity in STZ-induced diabetic rats (Wang et al., 2011). Further, Gao et al. (2007) reported that oleanolic acid, when administered in a dosage of 100 and 200 mg/kg body weight/day, for 40 days, showed a significant hypoglycemic effect in STZ-induced diabetic rats.

##### Bark

The methanolic extract of *Senna auriculata* (L.) Roxb. bark found effective in lowering the blood glucose level. After the administration of the bark extract to diabetic rats, it was observed that the blood glucose level reduced with a deviation of 80.9% when compared that on first day (Shiradkar et al., 2011). Moreover, Daisy and Jeeva Kani (2012) demonstrated the potent anti-diabetic effect of the methanolic extract of *Senna auriculata* (L.) Roxb. bark. The result was compared with hexane, ethyl acetate, and aqueous extracts where methanolic bark extract was found to be more effective in diabetic rats. Juan-Badaturuge et al. (2011) and Habtemariam (2013) reported the presence of rutin, quercetin, and kaempferol in the extracts of bark of *Senna auriculata* (L.) Roxb. Rutin and kaempferol have been reported to exhibit antihyperglycaemic and antioxidant activity in STZ-induced diabetic rats (Kamalakkannan and Prince, 2006; Govindasamy, 2020). Besides, quercetin has also been reported for antihyperglycemic potential due to its pleiotropic mechanisms. It showed the enhancement of insulin sensitivity, promotion of glycogen synthesis, improvement in insulin resistance by promoting its sensitization, and stimulation of pancreatic β-cell proliferation (Salehi et al., 2020). Therefore, the metabolites present in the bark may contribute to the antihyperglycemic action of the bark of *Senna auriculata* (L.) Roxb.

##### Seed

Jain and Sharma (1967) reported the antidiabetic activity of the aqueous extract of seed of *Senna auriculata* (L.) Roxb. The hypoglycemic role of the ethanolic seed extract in alloxan-induced diabetic rats has been demonstrated at a dose of 400 mg/kg body weight where the result was comparable with that of the standard drug, gliclazide. Also, the urine sugar was found absent in seed extract and gliclazide treated diabetic rats (Subramanian et al., 2011). Moreover, the ethanolic extract, aqueous extract, and petroleum ether fraction at a dose of 300 mg/kg body weight has reported for their significant (*p* < 0.001) blood-glucose-lowering activity in STZ-induced diabetic rats. The result was compared with the standard drug, metformin (250 mg/kg body weight) (Dama and Bhanoji Rao, 2011). Aruna and Roopa (2011) demonstrated the anti-diabetic potential of petroleum ether and ethyl acetate extract of *Senna auriculata* (L.) Roxb. seeds in alloxan-induced diabetic rats. It was found that both the extracts possess significant (*p* < 0.05) anti-diabetic activity when compared with the standard drug, tolbutamide (250 mg/kg body weight).

Seeds of *Senna auriculata* (L.) Roxb. is a rich source of antidiabetic metabolites like Linoleic acid, *n* -hexadecanoic acid, Oleic acid, Epicatechin, Procyanidin B1, Dodecanoic acid, Stearic acid, etc. (Suresh et al., 2007; Puranik et al., 2011; Raj et al., 2012). George et al. (2018) discussed the findings of Wu and colleagues’ pooled analysis (Wu et al., 2017), where it is recommended to take increased dietary intake of linoleic acid-rich vegetable foods for the prevention of diabetes. Similarly, a higher intake of linoleic acid resulted in better glycemic control and improved insulin sensitivity (Belury et al., 2018). Further, oleic acid also accounted for the prevention of Type 2 Diabetes Mellitus, where it was observed that oleic acid might have some metformin-like effects (Palomer et al., 2018). The consumption of epicatechin, a natural flavonoid found in the seeds of *Senna auriculata* (L.) Roxb. has been reported to reduce blood glucose levels in diabetic patients (Abdulkhaleq et al., 2017). Furthermore, procyanidin B1 (PB1) has a significant hypoglycemic action. Li et al. (2021) investigated the interaction mechanisms of PB1 with protein tyrosine phosphatase-1B (PTP1B), where a binding affinity of PTP1B to PB1 resulted in down-regulation of the expression level of PTP1B in insulin-resistant HepG2 cells. Similarly, the oral administration of graded doses of dodecanoic acid (125, 250, and 500 mg/kg) significantly reduced the fasting blood glucose level in a dose-dependent manner in hyperglycemic rats (Alex et al., 2020). Tsuchiya et al. (2013) demonstrated the role of stearic acid as a potent PTP1B inhibitor *in vitro*. It is suggested that stearic acid may enhance insulin receptor signaling by inhibiting the PTPB1 activity and promotes glucose uptake into adipocytes.

Although the preclinical data and pharmacological actions of various antidiabetic metabolites support each other, no clinical data is available so far. Further *in vivo* studies are expected to calibrate the antihyperglycemic role of several metabolites present in the seeds of *Senna auriculata* (L.) Roxb.

##### Root

Salma et al. (2021) demonstrated antihyperglycemic activity of methanolic extract of the root of *Senna auriculata* (L.) Roxb. in a high-fat diet-induced type 2 diabetes mellitus C57BL/6 mouse model. The methanolic extract (150 mg/kg body weight) could reduce the blood glucose level gradually in 8 weeks of the experiment. The results were similar when compared to a metformin-treated group of diabetic mice. The highest amount of total polyphenols was present in the methanolic extract of root than its aqueous, ethanolic, and chloroform extracts. Further, the HPLC profile of polyphenols showed that coumaric acid is present in the methanolic extract of the root, which attributed to the antihyperglycemic activity of *Senna auriculata* (L.) Roxb. (Salma et al., 2021). The p-Coumaric acid, the most abundant isomer of Coumaric acid, displayed a substantial increase in the enzyme activity and improved the glucose consumption by the hepatic tissues when administered orally in STZ-induced diabetic rats. It normalizes disturbed glucose metabolism by decreasing hepatic glucose production through insulin release. Besides, it exhibited antihyperglycemic activity by protecting β-cells of the pancreas (Amalan et al., 2016). Thus, the methanolic extract of the root of *Senna auriculata* (L.) Roxb. may have strong antihyperglycemic effects due to the presence of coumaric acid.

**FIGURE 4:**
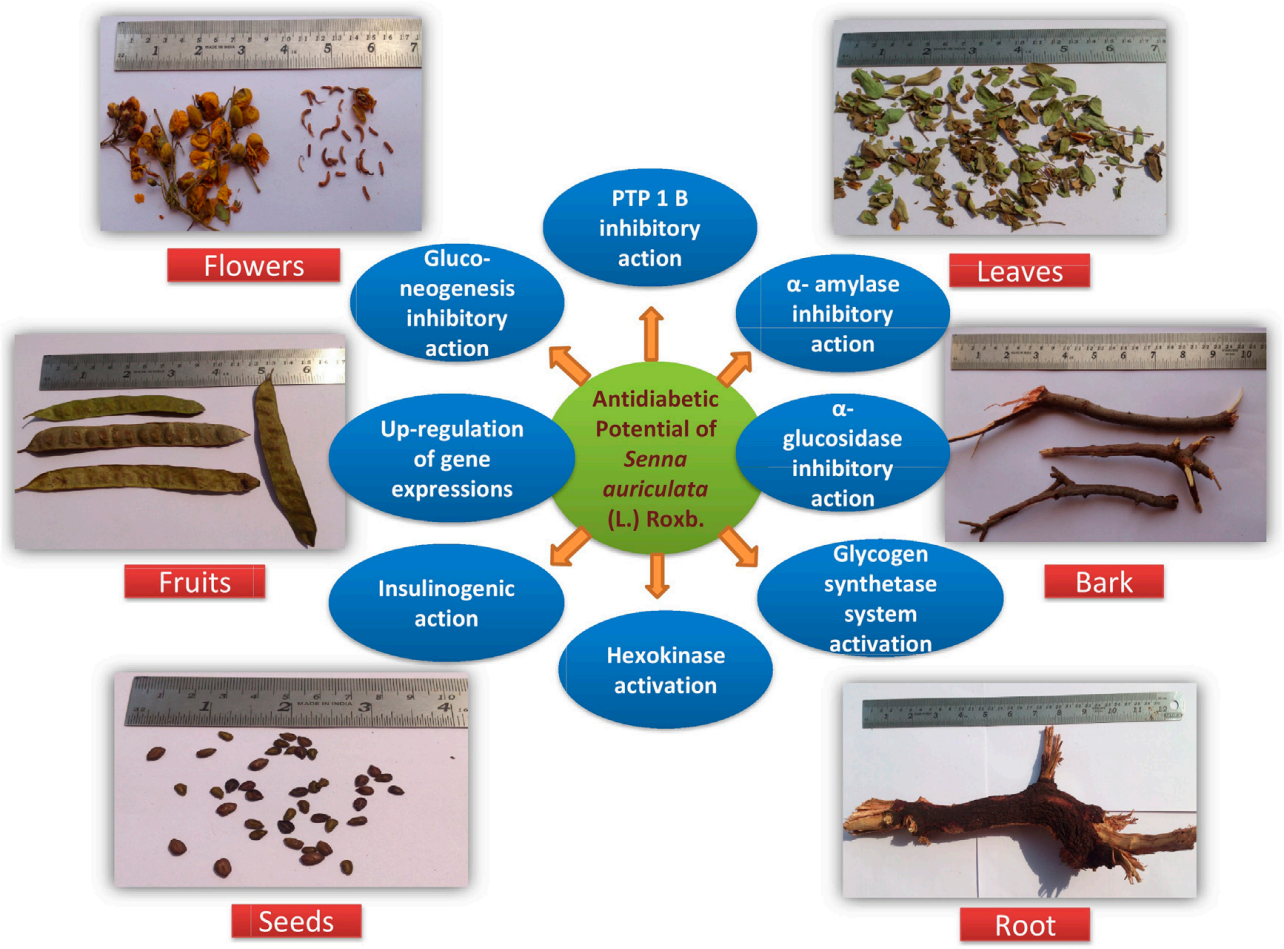
Pictorial presentation of different plant parts of *Senna auriculata* (L.) Roxb. and their anti-diabetic mode of actions.

Various antidiabetic metabolites present in the plant parts and their different extracts are responsible for the antihyperglycemic action. These metabolites may correct pathological changes, and further studies are needed to review their mode of action. The results were compared with available standard oral hypoglycemic agents and found significant at different levels. Altogether, all the botanical parts of *Senna auriculata* (L.) Roxb. exhibit antidiabetic activities and play a pivotal role in the correction of the pathological mechanism by regulating the metabolic pathways, enzymatic activities, and gene expressions (Figure 4).

#### Insulinogenic Action

Various plant parts of *Senna auriculata* (L.) Roxb. have been reported to possess an insulinogenic action. The different extracts stimulate the insulin secretion by increasing the number of islets and β-cells and it is evidenced with the increased amount of C-peptide and histological pancreatic sections. The administration of the aqueous leaf extract of *Senna auriculata* (L.) Roxb. demonstrated the insulinogenic action in mildly and severely diabetic rats. The C-peptide level was also found increased. Pancreatic sections have confirmed the increased number of islets and β-cells. The result was similar to that of the standard drug, glibenclamide (Gupta et al., 2010).

The aqueous flower extract of *Senna auriculata* (L.) Roxb. exhibited the antihyperglycemic action by increasing the level of insulin in diabetic rats which resulted into the increased uptake of blood glucose by peripheral tissue (Pari et al., 2001). Furthermore, the water-soluble fraction of ethanolic extract of *Senna auriculata* (L.) Roxb. flower was found significantly effective (*p* < 0.001) than its aqueous extract (*p* < 0.05) in reducing the blood glucose due to its insulinogenic action in alloxan-induced diabetic rats (Hakkim et al., 2007). Kalaivani et al. (2008) reported the possibility of regeneration of β-cells in *Senna auriculata* (L.) Roxb. leaves and flowers extracts administered diabetic rats. Moreover, oleanolic acid, an antidiabetic metabolite present in the aerial part of *Senna auriculata* (L.) Roxb. may directly improve insulin biosynthesis, secretion, and signaling by modulating the enzymes connected to insulin activities. It also protects the β-cells and preserves their functionality (Castellano et al., 2013). Similarly, Jyothi et al. (2012) reported the insulinogenic action of *Senna auriculata* (L.) Roxb. flowers.

To study insulin interaction in humans, erythrocytes have been used as a cellular model (Gambhir et al., 1978; DePirro et al., 1980; McElduff and Eastman, 1981; Ward and Harrison, 1986). Insulin binding is found to be decreased in diabetes mellitus (Kolterman et al., 1981; Olefsky and Kolterman, 1981). Pari et al. (2007) used the circulating erythrocytes to find out the insulin-receptor-binding effect of flower extract of *Senna auriculata* (L.) Roxb. in STZ-induced diabetic rats. It was found that the number of insulin receptors was increased on erythrocyte receptors membranes in STZ-induced diabetic control rats. The administration of flower extract resulted in increased total erythrocyte receptors membrane insulin binding sites as well as plasma insulin. *Senna auriculata* (L.) Roxb. flower extract stimulates insulin secretion and increases the number of insulin binding sites.

*Senna auriculata* (L.) Roxb. seed extract has also been reported to possess an insulinogenic action in alloxan-induced diabetic rats. The administration of seed extract resulted in increased insulin levels due to the activation of β-cells (Subramanian et al., 2011). Dodecanoic acid, also known as lauric acid, an antidiabetic metabolite, is present in the seed part of the *S. auriculata.* (L.) Roxb. (Suresh et al., 2007; Puranik et al., 2011; Raj et al., 2012). Alex et al. (2020) reported that dodecanoic acid (250 and 500 mg/kg body weight) stimulated the pancreatic β-cells to synthesize and secrete the insulin to maintain glucose homeostasis in a high-fat diet/STZ-induced type 2 diabetic rat models. The methanolic extract of *Senna auriculata* (L.) Roxb. bark potentiated the levels of insulin and C-peptide in diabetic rats. The activated remnant β-cells found in histological sections of the pancreas (Daisy and Jeeva Kani, 2012). Mohan et al. (2011) also reported the insulinogenic action of ethanolic extract of *S. auriculata* (L.) Roxb. leaf by its insulin release stimulatory effect in alloxan-induced diabetic rats.

The various extracts of botanical parts of *Senna auriculata* (L.) Roxb. demonstrated insulinogenic action. It is due to an increased number of β-cells and regeneration and activation of remnant β-cells. Moreover, the increased level of plasma insulin is evidenced with raised C-peptide level, a part of proinsulin, and a measure of insulin secretion. The metabolites and their pleiotropic mechanisms should also be studied extensively through *in vitro* and *in vivo* studies to know the insulinogenic action of *Senna auriculata* (L.) Roxb.

#### Role in Carbohydrate Metabolism and Regulation of Enzymatic Activities

Liver and pancreas ailments hamper the biochemical pathways in the human body and cause deranged glucose metabolism. The liver and pancreas play a vital role in governing carbohydrate metabolism. They maintain blood glucose and also regulate blood glucose supply to other organs.

##### Inhibition of Alpha-Amylase and Alpha-Glucosidase Enzymes

Starch and sucrose are the main components of carbohydrates. The enzyme alpha-amylase of saliva and pancreatic juice decomposes starch into oligosaccharides. The alpha-glucosidase enzyme catalyses the cleavage of glucose from disaccharides and oligosaccharides. Hence, the use of Alpha-glucosidase inhibitors is considered one of the effective treatments of diabetes mellitus as they retard digestion of both sucrose and starch.

The alpha-amylase inhibition activity of extract of bud and flowers of *Senna auriculata* (L.) Roxb. have been revealed where the bud extract showed higher inhibition activity compared to flower extract (Nambirajan et al., 2018). Also, the hydroalcoholic extract of aerial parts of *Senna auriculata* (L.) Roxb. showed the concentration-dependent alpha-amylase inhibition activity which found better than standard drug, acarbose (Mhetre et al., 2014).

The alpha-glucosidase enzyme plays a dominant role in the digestion of the sucrose and starch and its inhibition slows down the process of carbohydrate digestion. It is reported that *Senna auriculata* (L.) Roxb. bud extract and *Senna auriculata* (L.) Roxb. flower extract possess glucosidase inhibitory activity. The concentration of the bud and flower extracts shows a direct proportion to the inhibition of α glucosidase enzyme (Nambirajan et al., 2018). Alpha-glucosidase inhibitory activity of the hydroalcoholic extract of aerial parts of *Senna auriculata* (L.) Roxb. was found higher than the standard acarbose (Mhetre et al., 2014). Methanol extracts of dried flowers of *Senna auriculata* (L.) Roxb. were found to have a potential alpha-glucosidase inhibitory activity as compared to acarbose in rats. The ED_50_ of the methanol extracts of dried flowers (4.9 mg/kg) exhibited the antihyperglycemic effect as potent as that of standard drug, acarbose (ED_50_^−^ 3.1 mg/kg) in maltose loaded Sprague Dawley Rats (Abesundara et al., 2004).

The metabolites present in the Senna auriculata (L.) Roxb. extracts of acetone, ethanol, and water indicated the presence of flavonoids, tannins, reducing sugar, and saponins. The active components of the extracts compete with the substrate for binding to the active site of the enzyme and prevent the breaking down of oligosaccharides to disaccharides. Kwon et al. (2007a) reported that natural alpha-glucosidase inhibitors from plants inhibit alpha-glucosidase activity and can be potentially used as a safe and effective therapy for postprandial hyperglycemia. Previous studies on alpha-amylase and alpha-glucosidase inhibitors isolated from medicinal plants suggest that several potential inhibitors belong to the flavonoid class, which has features of inhibiting alpha-amylase and alpha-glucosidase activities (Kwon et al., 2007b). Senna auriculata (L.) Roxb. is a rich source of flavonoids such as kaempferol-3-O-rutinoside, kaempferol and quercetin (Juan-Badaturuge et al., 2011; Habtemariam, 2013). It was evident that kaempferol-3-O-rutinoside is a potent inhibitor of alpha-glucosidase *in vitro* and showed 8-folded activity than the standard drug, acarbose (Habtemariam, 2011).

Altogether, the effective inhibition of alpha-amylase and alpha-glucosidase might be due to the presence of enzyme inhibiting metabolites present in *Senna auriculata* (L.) Roxb. However, more *in vitro* and *in vivo* studies would be helpful to find out the pathways involved in the regulation of enzymatic activities.

##### Activation of Hexokinase

Enzymatic activity of hexokinase solely depends on insulin response and it is found affected in insulin-deficient diabetic rat liver (Gupta et al., 1997). *Senna auriculata* (L.) Roxb. is reported to play an important role in the activation of hexokinase in alloxan-induced diabetic rats which found resulted in increased glycolysis and glucose consumption for energy production (Krentz, 2003). The insulinogenic action of *Senna auriculata* (L.) Roxb. stimulates hexokinase activity which aids its role in glycolysis in all body tissues.

##### Inhibition of Glucose-6-Phosphatase and Fructose-1,6-Biphosphatase Enzymes

The role of Glucose-6-phosphatase and fructose-1,6-biphosphatase is also important in glucose homeostasis (Berg et al., 2001). These enzymes are known as gluconeogenic enzymes and insulin deficiency results in their activation in diabetes. It was found that the administration of *Senna auriculata* (L.) Roxb. effectively reduced the gluconeogenesis and resulted in decreased blood glucose level in diabetic rats. The augmented plasma insulin level by *Senna auriculata* (L.) Roxb. resulted in the reduced levels of glucose-6-phosphatase and fructose-1,6-biphosphatase enzymes in diabetic rats (Khader et al., 2017). Daisy and Jeeva Kani (2012) also reported the gluconeogenesis inhibitory action of *Senna auriculata* (L.) Roxb. bark extract.

##### Glycogen Synthesis

The healthy liver plays an important role in glycogen synthesis. It is the primary intracellular storage form of glucose and monitored by plasma insulin level and glycogen synthetase system (Garvey, 1992). The impaired capacity of the liver to synthesize glycogen was observed in diabetes due to lack of insulin. The dianthrone rich alcoholic flower extract of *Senna auriculata* (L.) Roxb. has demonstrated the increase in insulin secretion and activation of glycogen synthetase system which resulted in improved liver glycogen content (Khader et al., 2017).

Insulin deficiency in diabetes severely hampers the activities of glycolytic and gluconeogenic enzymes (Anderson and Stowring, 1973). Insulin is important for glucose uptake, phosphorylation of glucose and the glucose-6-phosphate entry into the pentose phosphate pathway in the liver (Ramachandran et al., 2003). The insulinogenic action of different extract of *Senna auriculata* (L.) Roxb. might be responsible for the modifications in enzymatic activities and reducing the blood glucose level (Kalaivani et al., 2008).

The flower extract of *Senna auriculata* (L.) Roxb. has also been reported for its salutary effect on hepatic enzymes involved in the carbohydrate mechanism. The flower extract (450 mg/kg body weight) was found significant when compared with the standard drug, glibenclamide (600 µg/kg body weight) in increasing the hexokinase activity and decreasing the gluconeogenic enzyme activity in STZ-induced diabetic rats. The increased plasma insulin level in flower extract-fed diabetic rats was responsible for the modulation of hepatic carbohydrate metabolic enzymes (Latha and Pari, 2003a; Latha and Pari, 2003b).

**FIGURE 5:**
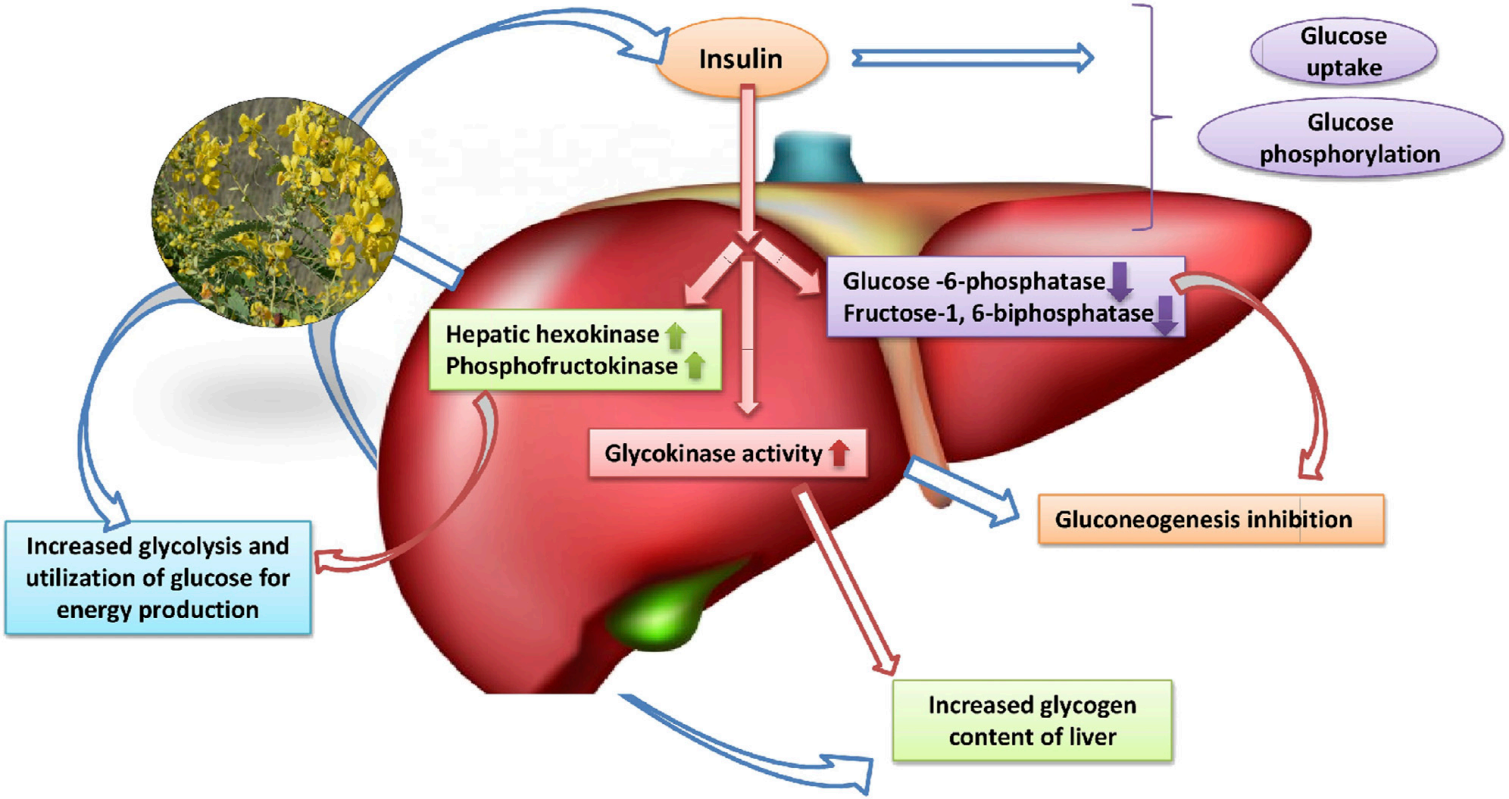
Diagrammatic presentation of the hepatic pathways and role of liver involved in the anti-diabetic action of *Senna auriculata* (L.) Roxb.

The liver glycogen level and glycogen synthetase system depend on adequate plasma insulin level. The water-soluble fraction of ethanol extract of flower of *Senna auriculata* (L.) Roxb. significantly (*p* < 0.001) elevated the levels of hepatic glycogen and glycogen synthase due to its insulinogenic effects (Hakkim et al., 2007). Similarly, glucokinase, one of the key enzymes in the liver, regulate the storage and disposal of glucose. The decreased enzymatic activity of glucokinase in STZ-induced diabetic rats was found elevated with *Senna auriculata* (L.) Roxb. bark extract treatment (Daisy and Jeeva Kani, 2012). Altogether, *Senna auriculata* (L.) Roxb. reveals its active role in the correction of the deranged carbohydrate metabolism by regulating the various enzymatic activities in the liver (Figure 5).

#### Regulation of Gene Expression in Liver

**FIGURE 6:**
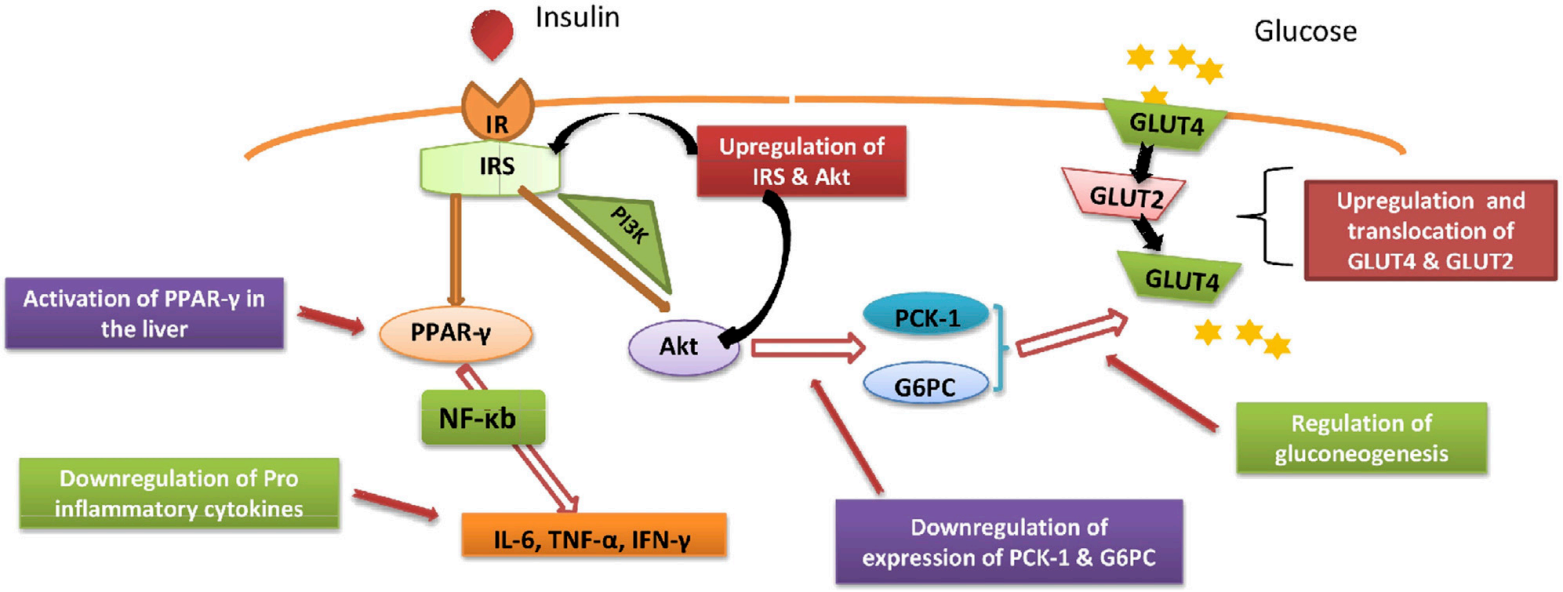
Schematic depiction of the possible cellular mechanism underlying Anti-diabetic Potential of *Senna auriculata* (L.) Roxb. in Type II DM.

IRS-2 gene arbitrates insulin activity through the PI3K-Akt pathway in the liver (Eckstein et al., 2017). Also, IRS-2 plays a major role in suppressing gluconeogenesis and apoptosis (Valverde et al., 2004). It is found that mice lacking IRS-2 have a better chance to develop diabetes (Kubota et al., 2000). The failure in the expression of IRS-2 has observed in diabetic individuals (Gunton et al., 2005). Besides, IRS-2 gene inactivation in the human will result in peripheral insulin resistance and absence of β cell expansion which may cause hyperglycaemia, diabetes and death. Treatment with *Senna auriculata* (L.) Roxb. bud extract showed the upregulation of the IRS-2 gene expression in the liver (Nambirajan et al., 2018). Also, polyphenols from *Senna auriculata* (L.) Roxb. flowers were found able to enhance IRS-2, glucose transporters, and Akt gene expression in livers of T2DM rats (Mohd Fauzi et al., 2017) (Figure 6).

#### Hepatoprotective Action

The liver is a vital organ involved in the metabolism of food and drugs in the human body. The various toxicants and chemicals in the form of drugs and food result in different liver ailments. *Senna auriculata* (L.) Roxb. is used in liver diseases, in the traditional systems of Indian medicine. The folk claims have been validated with different experimental works. The various extracts from the leaves and root of *Senna auriculata* (L.) Roxb. are reported to exert a hepatoprotective role in oxidative stress-induced, ethanol and anti-tubercular-drug induced, hepatotoxicity in rats (Rajagopal et al., 2003; Jaydeokar et al., 2014), and D-galactosamine (D-GalN)-induced cytotoxicity in mouse hepatocytes (Nakamura et al., 2014). The hepatoprotective effects of methanolic extract of leaf and revertible histopathological changes in the carbon tetrachloride-induced liver damage in Wistar albino rats (Dhanasekaran and Ganapathy, 2011) have been documented. The drug paracetamol-induced liver toxicity has also been found protected with the aqueous and methanolic extracts of the flowers of the *Senna auriculata* (L.) Roxb. (Chauhan et al., 2009).

The levels of AST and ALT were found raised in alloxan-induced diabetic rats. The oral administration of water-soluble fraction of ethanol extract of flower of *Senna auriculata* (L.) Roxb. could efficiently (*p* < 0.001) reduce the AST and ALT levels. Further, the increased values of acid phosphatase (ACP) and alkaline phosphatase (ALP) in alloxan-induced diabetic rats were also declined with the administration of water-soluble fraction of ethanol extract (Hakkim et al., 2007).

Taken together, these outcomes supports the traditional use of *Senna auriculata* (L.) Roxb. as a hepatoprotective agent. However, there is a need for clinical evidence to confirm and validate the folklore claims, as human studies have not been performed so far.

#### Prophylactic Action on Pancreatitis

The aqueous leaf extract of *Senna auriculata* (L.) Roxb. demonstrated the prophylactic effect on ethanol-induced pancreatitis in a rat model. A significant reduction in the increased pancreatic enzymes such as serum α-amylase and lipase was observed at a dose of 400 mg/kg body weight. Histopathological studies also revealed normal findings in extract-treated rats (Gupta et al., 2016).

### Antihyperlipidemic Action

Several phytochemical compounds have been isolated from different plant parts of *Senna auriculata* (L.) Roxb. The isolated compounds such as kaempferol-3-O-rutinoside, quercetin, rutin, and luteolin exhibit different pharmacological actions (Juan-Badaturuge et al., 2011). The antihyperlipidemic potential of crude extract and isolated compounds could be demonstrated by observing their pancreatic lipase inhibitory action. Among the isolated compounds from the aerial parts of *Senna auriculata* (L.) Roxb., kaempferol-3-O-rutinoside possesses most potential pancreatic lipase inhibitory action (IC_50_ = 2.9 ± 0.50 mM) than that of rutin, luteolin and quercetin. Moreover, the crude extract of *Senna auriculata* (L.) Roxb. demonstrated inhibition of pancreatic lipase at IC_50_ of 6.0 ± 1.0 mg/ml (Juan-Badaturuge et al., 2011; Habtemariam, 2013). Oleanolic acid, a metabolite present in the aerial part of *Senna auriculata* (L.) Roxb. (Juan-Badaturuge et al., 2011), when administered in the dosage of 100 and 200 mg/kg body weight/day for 40 days, showed improved lipid profile in STZ-induced diabetic rats (Gao et al., 2007). Similarly, Vijayaraj et al. (2013) also reported the antihyperlipidemic effects of different extracts of *Senna auriculata* (L.) Roxb.

The *Senna auriculata* (L.) Roxb. leaves extract exhibited hypolipidemic effects by reducing cholesterol, triglycerides and LDL levels and increasing the levels of HDL in diabetic rats. The atherogenic index was also found raised indicating the cardiovascular risk preventive role of *Senna auriculata* (L.) Roxb. (Gupta et al., 2009c). In another study, leaves extract of *Senna auriculata* (L.) Roxb. has shown its reversal effects on dyslipidemia and apolipoprotein B (Gupta et al., 2011).

Furthermore, the ethanolic extract of flowers of *Senna auriculata* (L.) Roxb. demonstrated antihyperlipidemic effects in Triton WR 1339 (300 mg/kg body weight) induced hyperlipidemia in male albino Wistar rats. The flower extract at dose 450 mg/kg body weight/day found more significant (*p* < 0.001) in reducing the total cholesterol, triglyceride, low-density lipoprotein (LDL) and very-low-density lipoprotein (VLDL) levels and increasing the levels of HDL than the standard drug, lovastatin. The antihyperlipidemic effects of ethanolic extract of *Senna auriculata* (L.) Roxb. flowers might be exhibited due to reduced cholesterol biosynthesis by HMG-CoA reductase inhibition in the liver or by up-regulation of LDL receptors in the liver which involve in hepatic cholesterol clearance (Oh et al., 2006; Vijayaraj et al., 2013).

**FIGURE 7:**
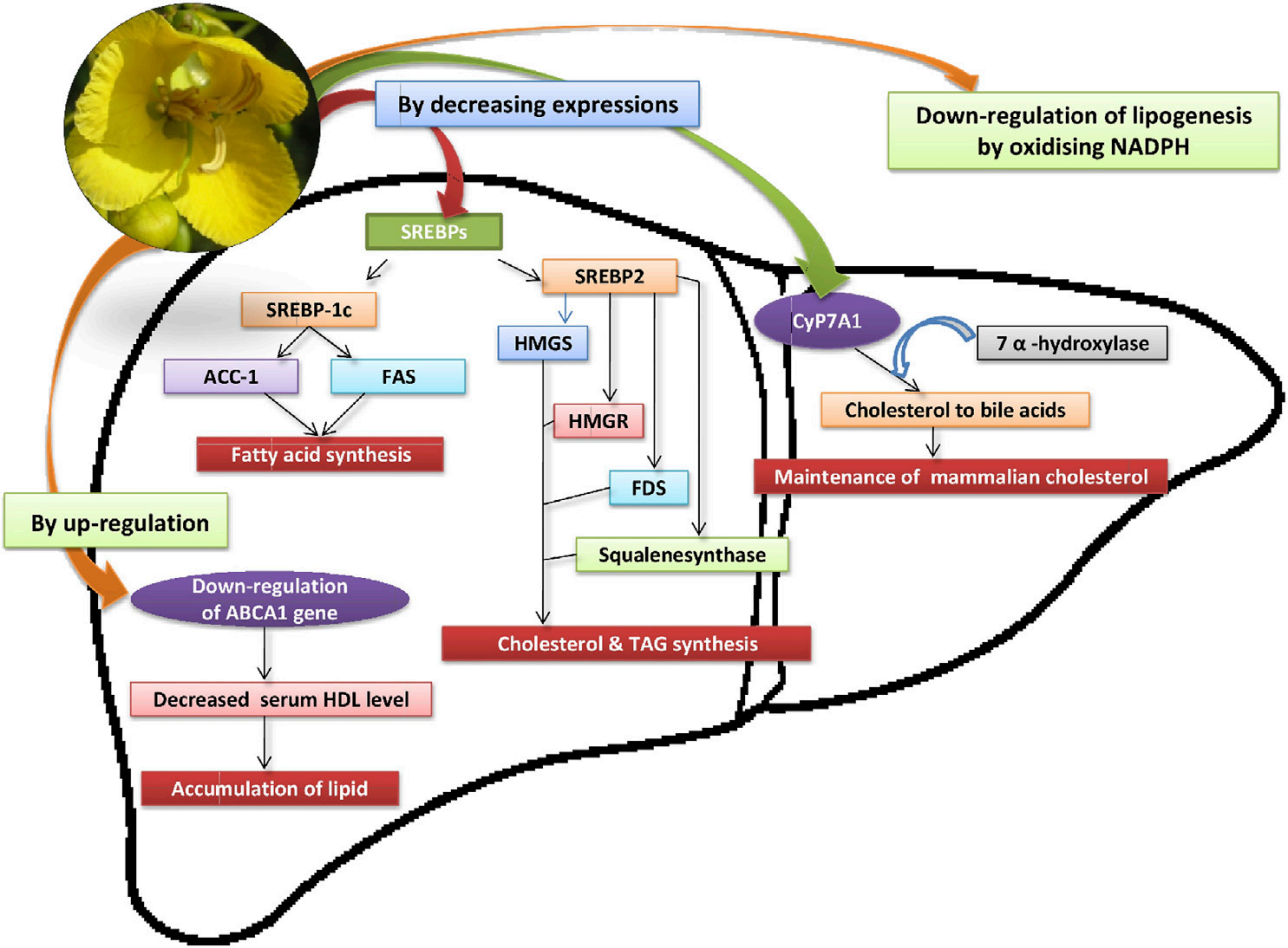
Effect of *Senna auriculata* (L.) Roxb. flower extract on gene and protein expression.

Similarly, in another study, the expression levels of the key genes involved in the cholesterol metabolism were examined in Triton WR-1339 induced hyperlipidemia in the male albino Wistar rats. It was found that the treatment with ethanol extract of *Senna auriculata* (L.) Roxb. flowers (300 mg/kg body weight) resulted in the reversal of altered protein and genes expression levels of HMGR, HMGS, SREBP-1c, ACC1, SREBP-2, CYP7A1, and ABCA1 to near-normal levels (Figure 7). The results were comparable with the standard drug, atorvastatin (Vijayakumar and Nachiappan, 2017).

Moreover, an aqueous extract of *Senna. auriculata* (L.) Roxb. flower may also capable to oxidize the NADPH, a co-factor in the lipid metabolism. The increased activity of glucose-6-phosphatase reduces NADP^+^ to NADPH by providing H^+^ which is helpful in the fat synthesis from carbohydrates. The administration of flower extract reduced glucose-6-phosphatase activity and produced high NADP^+^ from NADPH which ultimately resulted in down-regulation of lipogenesis (Pari and Latha, 2002).

Hakkim et al. (2007) compared the antihyperlipidemic potential of both aqueous extract and a water-soluble fraction of ethanol extract of *Senna auriculata* (L.) Roxb. flowers in alloxan-induced diabetic rats. It was found that the ethanol extract was exhibited significant (*p* < 0.001) reduction in the increased levels of triglycerides and total cholesterol than aqueous extract (*p* < 0.05) in diabetic rats. The restricted cholesterogenesis and reduced fatty acid synthesis were possible involved mechanism with the extracts of *Senna auriculata* (L.) Roxb. flowers in lowering of total cholesterol and triglycerides level in diabetic rats.

The seed extract of *Senna auriculata* (L.) Roxb. was also demonstrated antilipidemic activity where the significant reduction in cholesterol, triglycerides, LDL and increase in the HDL levels were observed in alloxan-induced diabetic rats (Subramanian et al., 2011). Furthermore, different extracts of seed such as aqueous, ethanolic, and pet ether and chloroform fractions were also ameliorated the altered levels of cholesterol and triglycerides in STZ-induced diabetic rats (Dama and Bhanoji Rao, 2011). Although it possesses antihyperlipidemic action, the effect in humans should be studied extensively.

### Antiatherosclerotic and Cardioprotective Action

The chronic diabetic state is often associated with cardiovascular risk development by atherosclerosis due to hypercholesterolemia, oxidative damage, activation of the inflammatory cascade and associated endothelial dysfunction and defective coagulation (Esterbauer et al., 1993; Dominiczak, 1998; Rask-Madsen and King, 2005). The oral administration of aqueous extract of *Senna auriculata* (L.) Roxb. leaves (400 mg/kg body weight) demonstrated potential anti-atherosclerotic action in STZ-induced diabetic rats. The extract suppressed lipid peroxidation and reduced the levels of oxidized LDL, soluble vascular cell adhesion molecule-1 and plasma fibrinogen. The increased serum nitric oxide level was also observed in extract-fed diabetic rats. It was also confirmed in histomorphological studies that the heart myocardium of extract-fed diabetic animals was showing normal morphology, whereas vacuolation was observed in the myocytes of the control diabetic rats (Gupta et al., 2011).

Lipid peroxidation inhibiting action, free radical scavenging activity, and anti-inflammatory action of different phytochemicals such as flavonoids, saponins, alkaloids and tannins etc. present in the aqueous extract of *Senna auriculata* (L.) Roxb. leaves may exhibit the significant anti-atherosclerotic and cardioprotective role in diabetic complications. For example, the leaves of *Senna auriculata* (L.) Roxb. is a rich source of squalene, a naturally occurring triterpenic hydrocarbon (Senthilkumar and Vijayakumari, 2012). Liu et al. (2018) demonstrated the effect of squalene on plasma and hepatic lipid levels of obese/diabetic KK-A^*y*^ mice and wild-type C57BL/6J mice. The administration of squalene was resulted in an increased HDL cholesterol level, an essential anti-atherosclerotic factor, while no significant difference was found in other lipid levels. Besides, the HDL level was found raised, especially in an obese/diabetes mouse model compared with normal mice. Hence, squalene may play a crucial role behind the anti-atherosclerotic and cardioprotective action of *Senna auriculata* (L.) Roxb. However, more research is needed to clarify the effect of extracts of leaves of *S. auriculata* (L.) Roxb. on the lipid metabolism and dynamics related to atherosclerosis. Overall, the different extracts of *Senna auriculata* (L.) Roxb. play an important role in the prevention of atherosclerosis due to their anti-hyperlipidemic effect (Pari et al., 2001). Only a few *in vivo* studies are available for the antiatherosclerotic effect of *Senna auriculata* (L.) Roxb. Besides, more preclinical and clinical studies should be conducted to validate its promising protective effect against atherosclerosis associated with metabolic diseases.

### Antifertility Action

In the early stages of pregnancy, the embryo nutrition and prevention of early abortion depend on the proper secretion of estrogen from corpus lutea. The methanolic extract of *Senna auriculata* (L.) Roxb. bark has demonstrated significant antifertility activity in experimental rats at doses of 100 and 200 mg/kg body weight. The oral administration of methanolic bark extract was continued for 7 days during the estrous stage of experimental rats. After dissecting the pregnant rats on day 10, it was observed that the number of corpus lutea reduced significantly, and there was an increased number of resorptions in the extract-fed rats. The effects were dose-dependent, where the extract at a dose of 200 mg/kg showed 100% antifertility activity. *Senna auriculata* (L.) Roxb. possesses potential antifertility activity, which may exhibit due to its antiestrogenic action (Shiradkar et al., 2011). The extract of the bark of *Senna auriculata* (L.) Roxb. is reported to have flavoinoids such as luteolin, kaempferol, and quercetin (Juan-Badaturuge et al., 2011; Habtemariam, 2013). Lu et al. (2012) reported luteolin, kaempferol, and quercetin as inhibitors of estrogen biosynthesis *in vivo*. Further studies are required to investigate the role of metabolites present in different extracts of *Senna auriculata* (L.) Roxb for its antiestrogenic and antifertility activity.

### Cytotoxic Activity

The various isolated compounds and extracts of medicinal plants have been reported for their anticancer effects and may act as novel chemopreventive agents in the management of various types of cancer (Aruna and Sivaramakrishnan, 1990; Graham et al., 2000; Moongkarndi et al., 2004). Many phytochemical compounds having anti-cancer effects such as flavonoids, procyanidins and triterpene glycosides have reported to be present in different parts of the *Senna auriculata* (L.) Roxb. plant and been isolated and evaluated for their cytotoxic actions (Ye et al., 1999; Sanghi et al., 2000; Nakamura et al., 2003; Kumaran and Karunakaran, 2007). An isolated compound, 4-(4-chlorobenzyl)-2,3,4,5,6,7-hexahydro-7-(2ethoxyphenyl)benzo[h][1,4,7]triazecin-8(1H)-one, from *Senna auriculata* (L.) Roxb. leaves has demonstrated cytotoxic effects on human colon cancer cell line HCT 15 and induced apoptosis mediated cell death. The cytotoxicity of the isolated compound was due to its high lipophilicity resulted in the loss of membrane integrity of the cancer cells. The compound was also found to cause membrane disintegration and it was confirmed with the significant release of lactate dehydrogenase (LDH) from damaged cell membrane as a result of its apoptosis (Esakkirajan et al., 2014). Similarly, Prasanna et al. (2009) reported the anti-cancer activity of ethanolic extract of *Senna auriculata* (L.) Roxb. leaves in human breast adenocarcinoma MCF-7 and human larynx carcinoma Hep-2 cell lines. The extract exhibited a dose-dependent anti-cancer activity with IC_50_ values of 400 µg in MCF-7 cells and 500 µg in Hep-2 cells. The inhibition of the growth of extract-treated MCF-7 and Hep-2 cell lines was observed due to nuclear fragmentation and condensation followed by apoptosis mediated inhibition of the proliferation of both the cells. In the same way, another isolated compound, 3-O-beta-D-xylopyranosides (triterpine glycosides), form *C. dahurica* (Tian et al., 2006), which also found present in *Senna auriculata* (L.) Roxb. (Sanghi et al., 2000) and *Actaea asiatica* (Gao et al., 2006), showed anti-cancer activity against HepG2 cell, and hepatoma cells (Tian et al., 2006), and MCF-7 cell line (Gao et al., 2006).

However, very few studies were undertaken to establish the cytotoxic effect of *Senna auriculata* (L.) Roxb., due to which it is pretty early to come to any conclusion for its therapeutic implications in cancer patients.

### Immunomodulatory Effect

The flower extract from *Senna auriculata* (L.) Roxb. showed significant immunomodulatory effect in aged rats. The administration of extract in aged rats at different doses was found to activate T cell immunity with increased T and B cell percentage accompanied by an enhanced proliferation of splenocytes in both resting and LPS-stimulated cells. The increased number of T cells was further supported by observing the elevated counts of CD4^+^, CD8^+^, and CD25^+^ regulatory cells. Furthermore, the supplementation of polyphenols decreased ROS production by neutrophils in response to phorbol myristate acetate (PMA) and *Escherichia coli* activation that could conceivably harm multiple biological systems in aged individuals (John et al., 2011).

Rutin, the major metabolite present in flower extract of *Senna auriculata* (L.) Roxb. possesses therapeutic activity and shows potential as analgesic, anti-inflammatory, organ-transplantation, and anticancer effects. A report indicated the protective effect of rutin on humoral and cellular immunity in rat model and caused a significant elevation in antibody titer and total antibody levels (Ganeshpurkar et al., 2017).Thus, we can propose the possible role of rutin and other polyphenols present in *Senna auriculata* (L.) Roxb. as immunomodulatory agent.

### Nephroprotective Activity

The nephroprotective activity of ethanolic extract of *Senna auriculata* (L.) Roxb. root was evaluated in cisplatin- and gentamicin-induced renal injury in experimental rats. It was observed that the root extract reduced elevated levels of blood urea and serum creatinine effectively at a dosage of 300 and 600 mg/kg body weight in the cisplatin model and a dose of 600 mg/kg body weight in the gentamicin model (Annie et al., 2005). The ethanolic root extract could demonstrate the nephroprotective activity in cisplatin- and gentamicin-induced renal injury in male albino rats due to its antioxidant and nitric oxide free-radical-scavenging effects.

### Antipyretic Activity

The fraction of ethanolic extract of *Senna auriculata* (L.) Roxb. leaves and flowers, at doses ranging from 250 to 600 mg/kg body weight, showed significant antipyretic activity in yeast-induced pyrexia in experimental rats. The effects were comparable to that of a standard drug, aspirin (Vedavathy and Rao, 1991).

### Antiviral Activity

The antiviral activity of methanolic extract of flowers of *Senna auriculata* (L.) Roxb. was investigated in different cell lines, such as HeLa, Vero, Crandell Reus feline kidney (CRFK), and HEL cell cultures. The methanolic flower extract showed the strongest antiviral activity against herpes simplex-1 and 2, and moderate activity against vaccinia, vesicular stomatitis, coxsackie, respiratory syncytial, feline corona, feline herpes, parainfluenza, reo-1, sindbis, and puntatoro viruses (Arthanari et al., 2013). The methanolic extract of *Senna auriculata* (L.) Roxb. flowers could be a vital source of anti-herpetic compounds possessing antiviral activity against the double-stranded DNA enveloped Herpes simplex virus (HSV -1 and 2).

### Antihelmintic Activity

The anthelmintic activity of methanolic and ethanolic extract of *Senna auriculata* (L.) Roxb. leaves against earthworm was investigated at the dose level of 20, 40, 60 mg/ml. The standard anthelmintic albendazole was used to compare the results. All the extracts showed the concentration-dependent anthelmintic property. *Senna auriculata* (L.) Roxb. leaves exhibited significant effects (*p* < 0.05) at the tested concentrations (20, 40, and 60 mg/ml) as determined by the paralysis and death time. Among all extracts, methanolic extract (40 and 60 mg/ml) was reported its efficacy at par in causing paralysis and death of earthworm at all concentrations (Chaudhary and Kumar, 2014). The anthelmintic property of *Senna auriculata* (L.) Roxb. may be attributed to the metabolites especially tannins and phenolic compounds. Tannins act by binding to free protein in the gastrointestinal tract of the host or glycoprotein on the cuticle of the parasite and phenolic compounds by uncoupling oxidative phosphorylation hinder the energy production in helminth parasites (Sreejith, et al., 2013; Athnasiadou, et al., 2001). Further, *in vivo* and *in vitro* studies are needed to determine the role of metabolites present in different extracts of *Senna auriculata* (L.) Roxb. and to verify the antihelmintic activity.

### Anti-Melanogenesis Activity

Wang et al. (2019) studied the effect of *Senna auriculata* (L.) Roxb. in the inhibition of melanogenesis in B16 melanoma 4A5 cells. The presence of auriculataosides A and B (phlegmacin-type anthracenone dimer glycosides) in *Senna auriculata* (L.) Roxb. indicated in inhibition of microphthalmia-associated transcription factor, tyrosinase, tyrosinase-related protein (TRP)-1, and TRP-2 protein expression. The methanolic extract in the concentration range of 1–100 µg/ml exhibited significant inhibition of melanogenesis. These dimer glycosides were isolated from the methanolic fraction, and their inhibitory activity were detected in the dose range of 0.03–0.3 μM.

### Antioxidant Activity

The various studies suggested the potency of *Senna auriculata* (L.) Roxb. as antioxidant agents in several assays such as ferric reducing antioxidant power (FRAP), 1,1-diphenyl-2-picrylhydrazyl (DPPH) free radical scavenging, hydroxyl radical scavenging, phosphomolybdenum reducing power, β-carotene bleaching assay, hydrogen peroxide radical scavenging, metal chelating activity, and deoxyribose degradation (Kolar et al., 2018). The antioxidant property of the *Senna auriculata* (L.) Roxb. measured by FRAP and DPPH assay was highest in flower extracts i.e. 161.5 mg AAE/g.

The methanolic and ethanolic extract of *Senna auriculata* (L.) Roxb. has potential free radical scavenging action in both 2,2′-azinobis-(3-ethylbenzothiazoline-6-sulfonicacid) (ABTS) and 1,1-diphenyl-2-picrylhydrazyl (DPPH) assays (Kumaran and Karunakaran, 2007). Jeyashanthi and Ashok (2010) studied that flower extract of *Senna auriculata* (L.) Roxb. significantly lowers the TBARS (thiobarbituric acid reactive substances), hydrogen peroxide, and conjugated dienes and exert a potential antioxidant activity. The extracts also increase the important antioxidant enzymes like glutathione, catalase, superoxide dismutase, ascorbic acid, and vitamin E level in rats (Jeyashanthi and Ashok, 2010). The treatment with the leaf extract of *Senna auriculata* (L.) Roxb. at 400 mg/kg exhibited significant decrease in serum levels of oxidized low-density lipoprotein (Ox LDL) and TBARS (Gupta et al., 2009c). The alcoholic extract of the aerial part of *Senna auriculata* (L.) Roxb. exhibited potent antioxidant activity when assessed by DPPH radical scavenging, lipid peroxidation, and reducing power analysis (Juan-Badaturuge et al., 2011).

The phosphomolybdenum assay involves the reduction of Mo (VI) to Mo (V) in the presence of antioxidant compound and subsequent formation of a green phosphate Mo (V) complex at acidic pH. The flower extracts (63.8 mg AAE/g) showed higher antioxidant activity of *Senna auriculata* (L.) Roxb. The highest ferrous ion chelating activity (90.05%) is reported in the extracts of flowers of *Senna auriculata* (L.) Roxb. (Kolar et al., 2018).

Oxidative stress is the main etiology behind many diseases and the antioxidant potential of *Senna auriculata* (L.) Roxb. along with above reported studies, supports its medicinal use in diabetes like metabolic diseases. The presence of polyphenols like rutin and kaempferol might be responsible for its free radical scavenging activity. Hence, these scientific evidence propose the possible therapeutic benefits of *Senna auriculata* (L.) Roxb. in various diseases.

### Antimutagenic Activity

The antimutagenic activity of ethyl acetate extract of *Senna auriculata* (L.) Roxb. in cyclophosphamide induced chromosomal damage in bone marrow cells of albino mice was studied by Panigrahy et al. (2011). At dose level of 100 and 200 mg/kg, it provides a significant protection (*p* < 0.05) against chromosomal aberration due to presence of flavonoids in ethyl acetate extract.

The concentration-dependent inhibitory effect of the methanolic extract of *Senna. auriculata* (L.) Roxb. bark on the mutagenicity of Acridine orange (AO) was studied at the concentration of 2.3, 11.4 and 22.8 µM. The probable mechanism of antimutagenic activity of methanolic extract of *Senna auriculata* (L.) Roxb. bark, could be due to presence of excellent scavengers of reactive oxygen species (ROS) like singlet oxygen and/or superoxide anion radical whichplay a central role in multistage mutagenesis and carcinogenesis (EFSA Scientific Committee, 2011; Deshpande et al., 2013a; Deshpande et al., 2013b).

### Antimicrobial Activity

*Senna auriculata* (L.) Roxb. exhibited the antimicrobial activity against *Escherichia coli*, *Salmonella typhi*, *Proteus mirabilis,* and *Klebsiella pneumoniae*. The studies on the antibacterial activity of alcoholic and aqueous extracts of flower of *Senna auriculata* (L.) Roxb. were demonstrated by using *Staphylococcus aureus*, *Enterococcus faecalis*, *Bacillus subtilis*, *Salmonella typhi*, *Salmonella paratyphi A*, *Escherichia coli*, *Proteus mirabilis*, *Pseudomonas aeruginosa*, *Klebsiella pneumoniae*, *Vibrio cholerae*, and *Shigella dysentrae*. The maximum activity was observed against all organisms except *Pseudomonas aeruginosa* and *Klebsiella pneumoniae*. The minimum inhibitory concentration ranged between 12.5 and 75 mg/ml depending on micro-organism and various extract. This study confirmed the antimicrobial activity of flower extract of *Senna auriculata* (L.) Roxb. and reported that it exhibits significant broad-spectrum activity against *Bacillus subtilis* and *Staphylococcus aureus* (Perumalsamy and Ignacimuthu, 2000).

Another study was performed to evaluate the antimicrobial activity of aerial parts of *Senna auriculata* (L.) Roxb. The chloroform extract of *Senna auriculata* (L.) Roxb. were shown to possess an antimicrobial activity against 2 g positive and 2 g-negative human pathogenic bacteria, viz. *Bacillus subtilis*, *Staphylococcus aureus*, *Pseudomonas aeruginosa*, *Escherichia coli* and fungus cultures such as *Candida albicans* and *Aspergillus niger*. The extract showed antibacterial activity at all concentrations selected, but only the extract with the concentration of 300 µg/ml showed maximum antibacterial activity against all the organisms except *Pseudomonas aeruginosa* which were comparable with the standard control, amikacin. The antifungal activity of chloroform extract of *Senna auriculata* (L.) Roxb. revealed significant effect against *Candida albicans* and *Aspergillus niger* with the net inhibition zone of 14 and 14 mm, respectively, at 300 µg/ml concentration, which is almost comparable with standard control, ketoconazole used as an antifungal agent (Raja et al., 2013; Gharge et al., 2017).

The saponins rich fraction of roots of *Senna auriculata* (L.) Roxb. was evaluated for antimicrobial activity against *P. vesicularis*, *Streptococcus faecalis*, *Aeromonas hydrophilia*, *Salmonella typhae*, *Staphylococcus cohni*, *Serratia ficaria*, and *E. coli* at concentration of 12.5, 25, 37.5 and 50 mg/ml. Antimicrobial activity of *Senna auriculata* (L.) Roxb. was carried out by well diffusion method. The result indicates the saponins rich fraction of roots of *Senna auriculata* (L.) Roxb. might be exploited as natural drug for the treatment of several infectious diseases caused by these organisms (Deshpande et al., 2013a; Deshpande et al., 2013b).

Further, Win and Min (2018) evaluated the antimicrobial activities of chloroform, acetone, methanol, ethyl acetate, and ethanol extracts of leaves of *Senna auriculata* (L.) Roxb. by using the agar-well diffusion method. The acetone extract exhibited maximum antimicrobial activity against *Pseudomonas aeruginosa*, *Bacillus pumalis*, and *Escherichia coli*, whereas the ethanol extract showed more antimicrobial activities against *Bacillus subtilis* and *Candida albicans*.

Thus, above data suggested that plant possess a good antimicrobial activity against various strains of pathogenic bacteria and fungus. The ethno-pharmacological utility of *Senna auriculata* (L.) Roxb. has been proven by cited studies but future prospects about the identification of active metabolites for the same is highly suggested, as their mechanism of action will determine its further therapeutic implications.

### Anti-Ulcer Activity

The anti-ulcer activity of methanolic extract of *Senna auriculata* (L.) Roxb. leaves (300 mg/kg body weight) was evaluated against pylorus ligation induced gastric ulcers, and the results were compared with the standard drug famotidine (10 mg/kg body weight) (Ahmed et al., 2010). It was observed that the *Senna auriculata* (L.) Roxb. leaf extract decreased the number of ulcers in pyloric ligated rats with a significant reduction in the gastric volume, free and total acidity, and ulcerative index.

### Anti-Inflammatory Activity

Mali et al. (2012) reported the role of *Senna auriculata* (L.) Roxb. leaf extracts viz. methanolic, ethyl acetate aqueous, and hydroalcoholic extracts in inflammation by using carrageenan induced paw edema. The highest inhibitory action in inflammation was exerted by methanolic extract. The methanolic extract showed significant anti-inflammatory potential at 6 h with percentage inhibition of 37% at 250 mg/kg and 31.63% at 500 mg/kg. This effect was attributed due to presence of alkaloids, flavonoids, tannins, and steroids. The ethyl acetate extract of *Senna auriculata* (L.) Roxb. at 250 & 500 mg/kg showed significant activity in second phase of inflammation induced by carrageenan with percentage inhibition of 34.16 and 30.79% respectively. The aqueous extract of *Senna auriculata* (L.) Roxb. (250 & 500 mg/kg) showed significant activity at 6 h with percentage inhibition of 31.06 and 30.62% respectively. The hydroalcoholic extract of *Senna auriculata* (L.) Roxb. in 250 & 500 mg/kg exerted significant activity at 6 h with percentage inhibition of 23.73 and 30.95% respectively. The standard indomethacin showed significant activity maximum at 6 h with percentage inhibition of 42.56%.

The presence of polyphenols in flower viz. rutin is responsible for the suppression of pro-inflammatory mediator’s release and expression of the inflammatory proteins such as adhesion molecules, COX, and NOS (Lilian et al., 1998; Habtemariam and Belai, 2018). Hence, the anti-inflammatory potential of *Senna auriculata* (L.) Roxb. has been proven its traditional uses in inflammation and needs further studies involving clinical subjects for its scientific and regulatory approval.

## Herbal Drug-Drug Interactions

The concurrent use of an herbal tea prepared from the flowers of the *Senna auriculata* (L.) Roxb. and theophylline performed a significant drug interaction. Their concurrent administration resulted in an increased steady-state level (32.5%) of drug theophylline (Thabrew et al., 2004). Similarly, they have also reported the potential drug interaction between *Senna auriculata* (L.) Roxb. tea and antiepileptic drug carbamazepine in experimental rats. The concurrent administration of herbal tea and carbamazepine in rats elevated (47.1%) the blood levels of the carbamazepine significantly than the levels in animals treated with carbamazepine alone. Therefore, the patients who have advised theophylline or carbamazepine should avoid the concurrent use of herbal tea prepared from *Senna auriculata* (L.) Roxb.flowers to influence the bioavailability of theophylline.

Puranik et al. (2011) reported safety pharmacology and pharmacokinetic herb-drug interaction studies of a hydro-alcoholic and supercritical extract of *Senna auriculata* (L.) Roxb. with metformin. Both these extracts were found safe at tested doses. However, the co-administration of technology-based supercritical extract (1,000 mg/kg) and metformin showed a significant decline (60%) in the absorption of metformin. The traditionally prepared hydro-alcoholic extract did not show any change in the pharmacokinetics of metformin. Furthermore, Elango et al. (2015) demonstrated the pharmacodynamic and pharmacokinetic interactions on co-administration of metformin and aqueous extract of *Senna auriculata* (L.) Roxb. leaves. The reduced dose of metformin (45 mg/kg) when combined with *Senna auriculata* (L.) Roxb. leaf extract (500 mg/kg) exhibited a similar blood-glucose-lowering effect of metformin (90 mg/kg) alone.

Further studies are needed to discover the possible pharmacodynamic and pharmacokinetic interactions between *Senna auriculata* (L.) Roxb. and various oral hypoglycemic agents to avoid serious adverse events in diabetic patients upon their concurrent use.

## Formulatons of *Senna auriculata* (L.) Roxb

*Senna auriculata* (L.) Roxb. is one of the main ingredients of many Ayurvedic and Siddha formulations available in the market. These formulations are commonly being used in diabetes, hyperlipidemia, obesity, and diabetic-associated co-morbidities. The formulations available in the market are enlisted in Table 2 **.**

**TABLE 2:** List of formulations in which *Senna auriculata* (L.) Roxb. is used as principal ingredient.

Sr. No.	Name of formulation	Ingredients	Indications	References
	Diasulin	*Senna auriculata* (L.) Roxb., *Curcuma longa* Linn., *Gymnema sylvestre* R. Br., *Coccinia indica* W. & A., *Momordica charantia* Linn., *Scoparia dulcis* Linn., *Syzygium cumini* Linn., *Trigonella foenumgraecum* Linn., *Tinospora cordifolia* Willd Miers.	Diabetes mellitus	Srivastava et al. (2012), Saravanan and Pari (2005)
2.	Sugnil	*Aristolochia bracteata* Retz., *Shorea roxburghii* G. Don, *Senna auriculata* (L.) Roxb., *Casearia esculanta* Roxb., *Coscinium fenestratum* (Gaertn) Colebr, *Curcuma longa* Linn., *Eugenia jambolana* Lam., *Gymnema sylvestre* R. Br., Triphala (three myrobalans)	Microvascular complications in Diabetes mellitus	Karthikeyan et al. (2011a), Karthikeyan et al. (2011b).
3.	Kalpa herbal tea	*Senna auriculata* (L.) Roxb.	Diabetes mellitus	Nille et al. (2016a)
4.	Avarai panchanga choornam	Equal quantities of fruits, leaves, roots, flowers, and bark of *Senna auriculata* (L.) Roxb.	Diabetes mellitus, Obesity	Latha and Pari, (2003a), Latha and Pari (2003b)
5.	Avarai kudineer	*Senna auriculata* (L.) Roxb., *C. fistula* Linn., *Syzygium cumini* Linn., *Olax scandens* Roxb., *Saussurea lappa* C.B. Clarke, *Terminalia arjuna*Roxb., *Cyperus rotundus* Linn.	Diabetes mellitus,Microbial and fungal infection	Rajalakshmi et al. (2018), Kumar et al. (2019), Prakash et al. (2014)
6.	Talapotaka churna	*Senna auriculata* (L.) Roxb., *Emblica officinalis* Gaertn, *Berberis aristata* DC., *Curcuma longa* Linn.	Diabetes mellitus, Obesity	Nille et al. (2016b)
7.	Avaram Poo	*Senna auriculata* (L.) Roxb. flower water extract	Diabetes mellitus	Sankhari (2019)
8.	Diazen	*Gymnema sylvestre* R. Br., *Momordia charantia* Linn, *Eugenia jambolana* Lam., *Tinospora cordifolia* Willd Miers, *Trigonella foenumgraecum* Linn., *Withania somnifera* Linn., *Senna auriculata* (L.) Roxb., *Aegle marmelos* Corr., *Azadirachta indica* A. Juss, *Curcuma longa* Linn.	Diabetes mellitus	Mishra and Mishra (2012)
9.	Hyponidd	*Pterocarpus marsupium* Roxb., *Gymnema sylvestre* R. Br., *Syzigium cumini* Linn., *Momordica charantia* Linn., *Enicostemma littorale* Blume, *Emblica*	Diabetes mellitus, Polycystic ovarian syndrome	Poongothai et al. (2002), Babu and Stanely Mainzen Prince (2004)
		*officianale* Gaertn, *Curcuma longa* Linn., *Melia*		
		*azadirachta* Linn., *Tinospora cordifolia* Willd Miers, *Senna auriculata* (L.) Roxb., Trivang Bhasma and Shilajit.		
10.	Dia Sakthi	*Centella asiatica* Linn.,*Curcuma longa* Linn., *Senna auriculata* (L.) Roxb., *Phyllanthus amarus* Schumach. &Thonn., *Tinospora cordifolia* Willd Miers, *Syzygium cumini* Linn., *Abrega Chendooram*, *Linga Chendooram*, Triphala Choorna	Diabetes mellitus	Nille and Reddy (2015)
11.	Dianex	*Aegle marmelos* Corr., *Gymnema sylvestre* R. Br., *Eugenia jambolana* Lam., *Momordica charantia* Linn., *Azadiracta indica* A. Juss, *Senna auriculata* (L.) Roxb., *Withania somnifera* Linn., *Curcuma longa* Linn.	Diabetes mellitus	Mutalik et al. (2003), Mutalik et al. (2005), Sudha et al. (2005)
12.	Diakyur	*Senna auriculata* (L.) Roxb.,*Cassia javanica* Linn., *Gymnema sylvestre* R. Br., *Mucuna pruriens* Linn., *Salacia reticulate* Linn., *Syzygium cumini* Linn., *Terminalia arjuna* Roxb.	Diabetes mellitus	Joshi et al. (2007)
13.	Diamed	*Azardirachta indica* A. Juss, *Senna auriculata* (L.) Roxb., *Momordica charantia* Linn.	Diabetes mellitus	Pari et al. (2001)
14.	Mersina	*Gymnema sylvestre* R. Br., *Momordica charantia* Linn., *Syzium cumini* Linn., *Phyllanthus emblica* Linn., *Trigonella foenumgraceum* Linn., *Coccinia indica* W. & A., *Tinospora cordifolia* Willd Miers, *Melia azadarichta*, Javakhar, *Senna auriculata* (L.) Roxb.	Diabetes mellitus	Belemkar and Veeranjaneyulu (2009)
15.	Byesukar	*Senna auriculata* (L.) Roxb., *Eugenia jambolana* Lam., *Thespesia populnea* Soland ex Correa	Diabetes mellitus	Guruvayoorappan and Sudha (2008)
16.	Diabkil	*Azardirachta indica* A. Juss, *Momordica charantia* Linn., *Tinospora cordifolia* Willd Miers, *Senna auriculata* (L.) Roxb., *Curcuma longa* Linn., *Terminalia arjuna* Roxb., *Piper nigrum* Linn., Shilajit, *Chlorophytum borivilianum* Sant., *Trigonella foenumgraecum* Linn., *Gymnema sylvestre* R. Br.	Diabetes mellitus	Grover and Bafna (2013)

### Toxicity Profile of *Senna auriculata* (L.) Roxb

Sabu and Subburaju (2002) carried out an acute toxicity study on aqueous extract of *Senna auriculata* (L.) Roxb. (leaf) on normal healthy albino Wistar rats at different doses (500, 1,000, 2,000, 5,000 mg/kg body weight). They showed that the extract did not produce any mortality up to the highest dose tested i.e. 5,000 mg/kg body weight. Also, animals did not exhibit any toxic signs like restlessness, respiratory depression, convulsion or coma.

In an acute toxicity study on albino Wistar male rats using ethanol extract of *Senna auriculata* (L.) Roxb. root suspended in acacia up to a dose 3,000 mg/kg body weight per oral observed that the alcoholic extract of *Senna auriculata* (L.) Roxb. did not produce any significant changes in the autonomic or behavioral changes including death during the observation period (Annie et al., 2005). Deshpande et al. (2013a) also carried out an acute toxicity study (OECD 423 guideline) at different dose levels of 5, 50, 300, and 2,000 mg/kg of ethyl acetate extract of roots of *Senna auriculata* (L.) Roxb. The rats did not show any toxic effects as well as any significant variation in their behavior. Acute toxicity studies of ethanol and aqueous extract of *Senna auriculata* (L.) Roxb. flowers on healthy adult male albino rats at a dose of 100, 500, 1,000, and 3,000 mg/kg body weight indicated the non-toxic nature of extracts in terms of mortality (Hakkim et al., 2007).

Gupta et al. (2009a) studied the toxicity of aqueous extract of *Senna auriculata* (L.) Roxb. leaves at a dose of 1,000 and 2,000 mg/kg body weight per oral once a daily for 3 weeks. The rats treated with 1,000 and 2,000 mg/kg doses of extract did not show any drug-induced physical signs of toxicity during the complete experimental period and no mortality was noted.

## Clinical Studies

Nille et al. (2018) evaluated the antidiabetic potential of *Talapotaka churna* (4 g TID), a polyherbal formulation containing *Senna auriculata* (L.) Roxb. as a major ingredient, in Type II Diabetes Mellitus patients. The results were compared with the standard drug, glimepiride (1 mg BD). In the 2 months of the clinical trial, *Talapotaka churna* improved the symptoms of diabetes such as polyurea, polyphagia, and polydipsia with effective reduction (*p* < 0.05) in fasting and postprandial blood glucose levels. Further, a significant (*p* < 0.01) improvement was reported in disturbed biochemical parameters such as serum creatinine, SGOT, SGPT, and lipid profile.

Similarly, the aqueous extract of *Senna auriculata* (L.) Roxb. flowers demonstrated a potential anti-hyperglycemic effect in pre-diagnosed type-2 diabetes mellitus subjects. A significant result (*p* < 0.001) was observed in 30 days of oral administration of flower extract with the effective reduction in fasting as well as postprandial blood glucose level (Sankhari, 2019).

Very little clinical data is available, and further extensive clinical studies are needed to support the experimental outcomes of *in vivo* studies and propose the antidiabetic potential of *Senna auriculata* (L.) Roxb. in human participants.

## Perspectives and Future Directions

The present review provides comprehensive data, relevant ethnomedicinal uses, details of metabolites, pharmacological activities of crude and various extracts along with pure compounds, marketed formulations, and its safety profile. Investigations on extracts and metabolites of *Senna auriculata* (L.) Roxb. provides a scientific evidences to explore the clinical implications as antidiabetic, antioxidant, anti-inflammatory, antihyperlipidemic, hepatoprotective, nephroprotective, cardioprotective, anti-atherosclerotic, anticancer, antimutagenic, antimicrobial, antiulcer, antipyretic, anthelmintic, immunomodulatory, antifertility, and anti-melanogenesis potentials with different underlying signaling pathways. The biological potential and mechanisms of action of many metabolites are need to be scientifically investigated for their molecular modes of action and bioactivities. It could provide a lead for further advancement into therapeutics. Well-organized, well-designed, and multicentric clinical studies should be warranted to evaluate the clinical efficacy and safety of the standardized extracts of *Senna auriculata* (L.) Roxb. carrying pharmacologically active metabolites. There are limited data from clinical reports on diabetes and associated complications. Based on available evidence data, it is advised that *Senna auriculata* (L.) Roxb. could be used as an adjunct to the current therapy for diabetes and other metabolic disorders.

## Conclusion

Though the available experimental evidence insinuates the therapeutic potential of *Senna auriculata* (L.) Roxb., to date, it's an unexploited plant species in clinical practice. The reason is the scattered experimental data and scarcity of clinical evidence. The present review focuses on addressing the safe pharmacological actions of *Senna auriculat*a (L.) Roxb. on multiple pathways involved in the pathogenesis of diabetes and various other diseases, which also indulges its ethnomedicinal uses in diabetes mellitus. Also, the extensive animal studies involved different extracts of all the botanical parts of *Senna auriculata* (L.) Roxb., which provides the limelight for the traditional claims and its medicinal uses in various ailments in folkloric practices. The metabolites present in the *Senna auriculata* (L.) Roxb. provide an ultimate scope for its wide acceptance in the scientific community to discover and produce synthetic molecules as an adjunct to the current therapy in NCDs. Its commercial cultivation and safety pharmacology aspects will contribute to the national economy and would be helpful to reduce the burden of NCDs with its medicinal uses.
